# Enhancing transcriptome expression quantification through accurate assignment of long RNA sequencing reads with TranSigner

**DOI:** 10.1186/s13059-025-03723-2

**Published:** 2025-08-28

**Authors:** Hyun Joo Ji, Mihaela Pertea

**Affiliations:** 1 Center for Computational Biology, Johns Hopkins University, Baltimore, MD USA; 2 Department of Computer Science, Johns Hopkins University, Baltimore, MD USA; 3 Department of Biomedical Engineering, Johns Hopkins University, Baltimore, MD USA

**Keywords:** Long-read RNA sequencing, Transcriptomics, Expression quantification

## Abstract

Long-read RNA sequencing captures transcripts at full lengths, but existing methods for transcriptome profiling using long-read data often produce inconsistent transcript identification and quantification results. Here, we introduce TranSigner, a tool designed to provide read-level support for transcripts in a given transcriptome. TranSigner consists of three modules: read alignment to transcripts, computation of read-to-transcript compatibility scores, and a guided expectation–maximization algorithm to assign reads to transcripts and estimate their abundances. Using simulated and experimental data from three well-studied organisms—*Homo sapiens*, *Arabidopsis thaliana*, and *Mus musculus*—we show that TranSigner achieves accurate read assignments and abundance estimates.

**Supplementary Information:**

The online version contains supplementary material available at 10.1186/s13059-025-03723-2.

## Background

Long-read RNA sequencing (RNA-seq) represents a remarkable advancement towards achieving full-length sequencing of transcripts, offering novel insights into transcriptomes previously characterized only with short reads. Short-read RNA-seq data has limitations in several applications such as transcript assembly, primarily due to its fragmented nature and inherent biases (e.g., GC content, amplification) that add noise to downstream analyses [1–3]. Long-read sequencing technologies address some of these limitations by substantially increasing the read lengths, enabling the possibility of capturing an entire transcript within a single read. However, long-read sequencing introduces its own set of challenges, with protocol- and platform-specific variations adding further complexity for researchers working with this type of data [4, 5]. For instance, Oxford Nanopore Technologies (ONT) direct RNA-seq reads are reported to be more error-prone, with a median error rate consistently exceeding 10%, compared to typical Illumina short reads, which often achieve error rates closer to 0.1% [6, 7].

Despite their potential, the full capabilities of long-read RNA-seq remain untapped due to the limited inventory of tools optimized for analyzing long-read data. Although tools such as FLAIR [8], Bambu [9], ESPRESSO [10], IsoQuant [11], and StringTie [12] are designed to characterize transcriptomes from long-read RNA-seq data, their results often lack agreement in identifying which transcripts are present and their corresponding abundances in a given sample [8–10, 13]. One way to address uncertainties in transcriptome assemblies is by assigning specific long reads to transcripts. This allows for a more in-depth evaluation of the read-level support for transcripts, as opposed to relying on read counts only. Given read-to-transcript assignments, transcripts can be directly associated with a distribution of supporting read lengths, quality scores, alignment positions, and more. These expanded sets of features can be used to derive a more confident set of transcripts and improve the accuracy of transcript abundance estimates.

Several existing tools, including FLAIR, Bambu, and IsoQuant, offer some form of read-to-transcript assignment. However, this functionality is integrated into larger pipelines and cannot be executed independently. Additionally, there are inconsistencies in how these assignments are presented. For example, Bambu reports whether a read matches a complete or partial exon junction of a transcript, while FLAIR provides a map linking transcripts to list of reads. Furthermore, when a read is assigned to multiple transcripts, tools often do not specify how the read fraction is distributed, preventing users from making quantitative assessments, such as ignoring assignments with sufficiently low read fractions. A standalone tool capable of performing read assignments on any input transcriptome would allow users to investigate any transcriptome of their choice, without being constrained by upstream tools, which might prioritize transcriptome identification. However, this need is largely unmet, with only a few recent methods, such as NanoCount [3, 14, 15] and Oarfish [3, 14, 15], attempting to address it.

Here we introduce TranSigner, a novel method for accurately assigning long RNA-seq reads to any given transcriptome while also achieving state-of-the-art accuracy in transcript abundance estimation. TranSigner first performs non-spliced alignment of long RNA-seq reads to the input transcriptome using minimap2 [16, 17]. It then extracts specific features from the alignment results to compute compatibility scores for each read and transcript pair, which indicate the likelihood of a read to originate from a specific transcript. TranSigner then employs an expectation–maximization (EM) algorithm to iteratively assign read fractions to transcripts and gradually derive maximum likelihood (ML) estimates for both read-to-transcript assignments and transcript abundances. We show that incorporating alignment-derived features during the E-step updates enables TranSigner to achieve highly accurate read-to-transcript mappings and, consequently, improve transcript abundance estimates as well. Among software capable of reporting read assignments, TranSigner stands out for its highly accurate read-to-transcript mappings.

## Results

### Simulated data performance

We first compared TranSigner against five other quantification-only or quantification-focused tools: NanoCount, Oarfish, Bambu, IsoQuant, and FLAIR. For tools that perform transcript identification (or assembly) prior to quantification (i.e., Bambu, IsoQuant, and FLAIR), we disabled this feature to ensure a direct comparison of their quantification abilities. We benchmarked all six tools using five sets of simulated ONT reads: three sets of direct RNA reads and two sets of cDNA reads. NanoCount was benchmarked twice, with and without its alignment filters. TranSigner and Oarfish were also benchmarked twice, with their coverage-usage options enabled (i.e., –psw flag for TranSigner and –model-coverage flag for Oarfish). Note that by default, TranSigner uses a simple algorithm to remove low-compatibility relations between reads and transcripts by dropping read-transcript pairs where only a small fraction of the read is assigned to the transcript, immediately after the first E-step update of its EM algorithm (see Methods). Inspired by Jousheghanis et al., we have also implemented a mode in which a “position-specific weights” model is built from the observed alignments to estimate the likelihood that a read covers specific base positions in the transcript. This mode is described in detail in the Methods section and can be activated using the –psw flag when running TranSigner.

The reads were simulated from protein-coding and long non-coding transcripts in the GRCh38 RefSeq annotation (release 110). Each tool was then benchmarked on the simulated reads, with the same RefSeq annotation provided as the target transcriptome (see Methods for a detailed description of the simulation). For simplicity, we will refer to the transcripts from which the reads were simulated as the origin transcripts. We evaluated each tool’s abundance estimates against the expected read counts of ground truth transcripts using three metrics: Spearman’s correlation coefficients (SCCs) and root mean squared errors (RMSEs) for raw read counts, as well as Pearson’s correlation coefficients (PCCs) for log-transformed read counts. SCC is useful for assessing whether the relative ranks of read counts are correctly estimated, while PCC and RMSE provide insights into the accuracy of the raw estimates. Raw read counts are important in long RNA-seq data analysis, as downstream analyses often involve count-based filtering and other post-processing decisions [18].

**Fig. 1:**
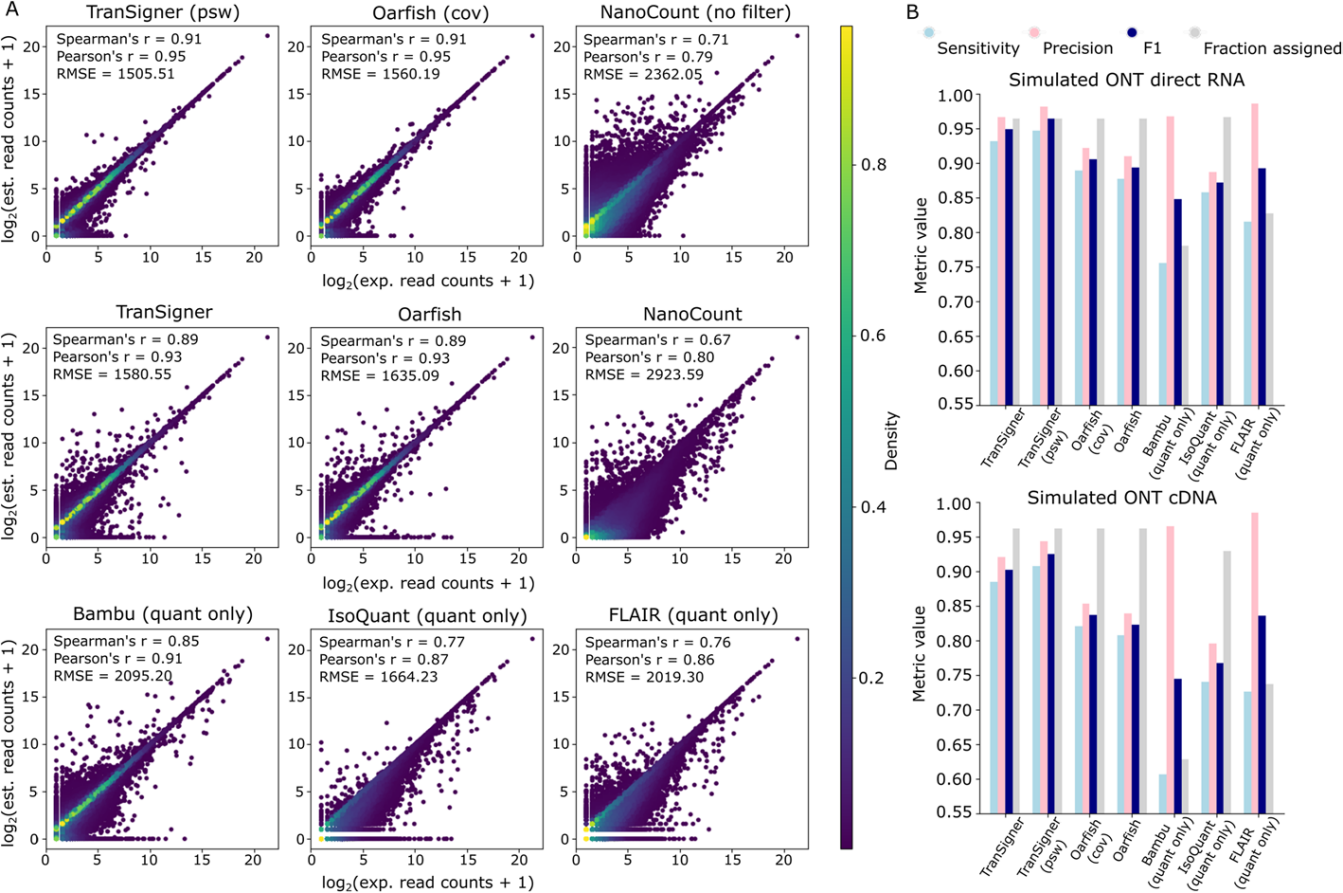
Comparison of read count estimates and read assignments across tools using simulated ONT RNA-seq data. **A** Correlation scatter plots comparing expected read counts to the read count estimates outputted by TranSigner, Oarfish, NanoCount, Bambu, IsoQuant, and FLAIR using the full RefSeq annotation and a set of simulated ONT direct RNA-seq reads. Each dot in the scatter plot represents a single transcript, and the plots include the corresponding SCC, PCC, and RMSE values. NanoCount (no filter) refers to the results obtained by running NanoCount without its alignment filters. TranSigner (psw) refers to results obtained with the –psw flag enabled during TranSigner runs. Oarfish (cov) refers to results obtained with the –model-coverage flag enabled during Oarfish runs. Bambu, IsoQuant, and FLAIR were run with their transcript identification features disabled, labeled as (quant only). **B** Evaluation of the read assignments generated by TranSigner, Oarfish, Bambu, IsoQuant, and FLAIR using the full RefSeq annotation on simulated ONT direct RNA data sets (top) and cDNA data sets (bottom)

When the full RefSeq annotation was provided as input to all tools, TranSigner (psw) and Oarfish (cov), representing the configurations using the –psw and –model-coverage flags, respectively, achieved the highest average correlation between abundance estimates and the ground truth in both simulated ONT direct RNA and cDNA data (see Fig. 1A, Table 1, and Additional file 1: Table S1). While both tools performed exceptionally well in correlation metrics, TranSigner outperformed Oarfish in terms of RMSE, achieving a lower average RMSE (≈1504) compared to Oarfish (≈1559) in simulated direct RNA data, a trend that was consistent in simulated cDNA data (see Additional file 1: Table S1). The next best performers were Oarfish and TranSigner in their default modes (i.e., without using coverage information). While Oarfish obtained slightly higher correlation values, it also exhibited a higher RMSE compared to TranSigner (see Additional file 1: Table S1). Additionally, Fig. 1A, which displays results from one dataset typical of all simulated data, shows a higher concentration of points along the diagonal in the scatter plots of TranSigner and Oarfish. This trend is less pronounced in the plots of the other tools. Specifically, the scatter plots for NanoCount, IsoQuant, and FLAIR show accumulations of points well below the diagonal, indicating their tendencies to underestimate read counts. While Bambu did not show signs of underestimation, its scatter plot displayed a large cluster of points near the origin along the diagonal line, suggesting a lack of correlation for low- or medium-expressed transcripts. Moreover, Bambu and Oarfish had relatively high standard deviation (SD) values of ≈507 and ≈45, respectively, for their RMSE values across the direct RNA read sets, a trend not observed in other tools (see Table 1). NanoCount showed an improvement in all metric values when its alignment filters were removed during testing with direct RNA reads. However, the opposite trend was observed with cDNA data, where NanoCount’s metric values were higher when the filters were enabled (see Additional file 1: Table S1).

**Table 1:** Average SCC, PCC, and RMSE metric values evaluating the transcript abundances estimated by TranSigner, Oarfish, NanoCount, Bambu, IsoQuant, and FLAIR when the full RefSeq annotation is provided. Average were taken across three sets of simulated ONT direct RNA sequencing reads

	**SCC**		**PCC**		**RMSE**	
	**Mean**	**SD**	**Mean**	**SD**	**Mean**	**SD**
**TranSigner**	0.89	0.00029	0.93	0.00030	1579.68	0.85
**TranSigner (psw)**	0.91	0.0013	0.95	0.00070	1504.10	1.54
**Oarfish (cov)**	0.91	0.0014	0.95	0.00068	1559.05	1.51
**Oarfish**	0.88	0.019	0.92	0.012	1634.37	1.39
**NanoCount**	0.67	0.0015	0.80	0.0010	2924.77	1.49
**NanoCount (no filter)**	0.71	0.00049	0.79	0.00067	2360.68	4.13
**Bambu (quant-only)**	0.85	0.0031	0.91	0.0021	2411.93	507.08
**IsoQuant (quant-only)**	0.78	0.0033	0.87	0.0011	1663.45	1.32
**FLAIR (quant-only)**	0.76	0.0020	0.86	0.00068	2017.29	1.95

We then compared the read assignment accuracies of TranSigner, Oarfish, Bambu, IsoQuant, and FLAIR when provided with the full RefSeq annotation. Performance was evaluated using recall, precision, and F1 scores, calculated based on the number of correctly versus incorrectly assigned reads (see Methods). Note that Bambu and IsoQuant do not provide read fraction distributions in cases of assignment ambiguity. Instead, they record a read as belonging to multiple transcripts. To evaluate them, we evenly split the read among the transcripts. However, we want to clarify that these programs were not specifically designed to provide read assignments. As shown in Fig. 1B, Bambu and FLAIR achieved superior precision values compared to their competitors. However, their poor sensitivity resulted in lower F1 scores. In contrast, both TranSigner (with or without employing position-specific weights) and Oarfish showed strong sensitivity and precision, positioning them as the top performers in both simulated direct RNA and cDNA data. TranSigner achieved the highest F1 scores across all tools.

We also tallied the total number of assigned reads and computed the assignment fraction by dividing these values by the total number of reads in each dataset. This analysis revealed that Bambu and FLAIR consistently assigned fewer than 80% of all available reads, potentially explaining their high precision values. In comparison, Oarfish, TranSigner, and IsoQuant all each achieved an assignment rate above 90% for the simulated reads.

Even for extensively studied species, gene annotation catalogs are often incomplete, missing both potential gene loci and many transcript isoforms [19, 20]. This incompleteness poses a significant challenge for accurate transcript quantification and explains why most long-read processing tools prioritize transcript identification before quantification. Providing only the origin transcripts as input, compared to the full reference annotation, improved correlation values and read assignments across most tools and simulated datasets (see Additional file 1: Tables S1 and S2). The exceptions were Bambu and NanoCount, which showed a slight reduction in abundance correlation values for simulated ONT direct RNA datasets. Bambu also experienced a minor drop in precision for read assignments; however, its read assignment fraction increased (see Additional file 2: Fig. S1 and Additional file 1: Table S2). This finding supports the intuition that limiting the input to origin transcripts reduces ambiguity in both read assignment and abundance estimation. In typical use cases, where the full set of expressed transcripts is not known a priori, these tasks become considerably harder. This emphasizes the importance of upstream steps, such as transcript identification or annotation refinement, which directly influence the reliability of downstream analyses.

Since the accuracy of the input transcriptome is critical for reliable transcript quantification, we next evaluated the performance of various transcriptome identification tools in achieving this. Obtaining an accurate transcriptome remains a challenging problem, with different tools showing varying levels of accuracies and reliance on the input reference annotation to guide the process. Using the same simulated ONT data sets (3 direct RNA, 2 cDNA) as before, we evaluated several transcript identification methods for their ability to handle incompleteness in the input guide annotations. To assess this, we randomly sampled the full RefSeq annotation to include varying percentages—between 0 and 100% with increments of 5%—of the origin transcripts and provided the resulting annotations as guides. This random sampling was repeated three times for every read set. We then run the tools under evaluation on each set of reads, using a different guide annotation each time. Due to this high number of iterations, speed was a major factor in determining which tools could be included in our comparison. Bambu, StringTie, and FLAIR demonstrated reasonable run times (1 h maximum), whereas IsoQuant and ESPRESSO required over 6 h and more than 24 h, respectively, to process a single set of simulated reads, even in multi-threaded mode. Consequently, we had to limit the number of evaluations for IsoQuant, ultimately running it only on the full RefSeq annotation, the 0% and 50% complete guide annotations, and in de novo mode.

**Fig. 2:**
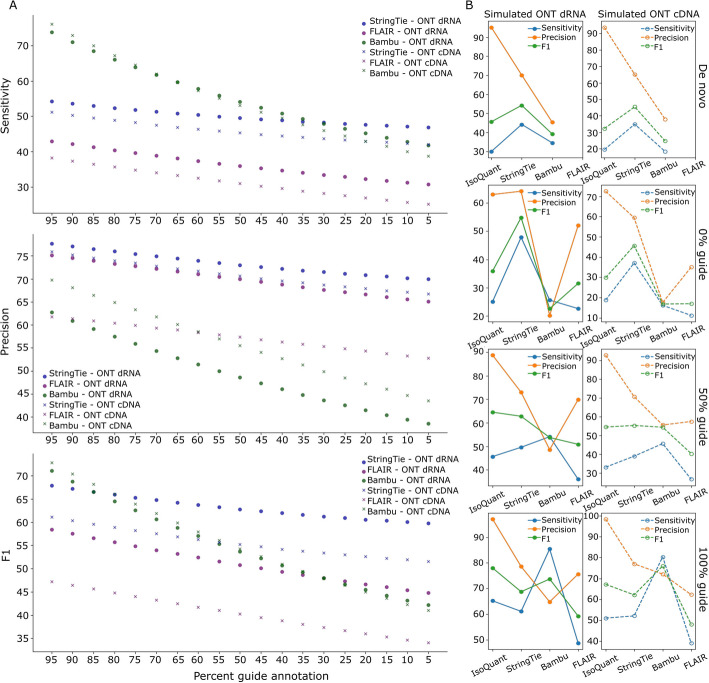
**A** Long-read assembly accuracies of StringTie, FLAIR, and Bambu with varying percentages (95% to 5%) of randomly sampled origin transcripts provided in the input guide annotation. Mean values for all three metrics—sensitivity, precision, and F1—across ONT direct RNA and cDNA datasets are shown as circles and crosses, respectively. **B** Average sensitivity, precision, and F1 values achieved by IsoQuant, StringTie, Bambu, and FLAIR when 100% (i.e., full), 50%, and 0% guide annotations are provided. The results for when IsoQuant, StringTie, and Bambu are run in their de novo modes are also shown. The left column shows the mean metric values in simulated ONT direct RNA data and the right column shows those in simulated ONT cDNA data

The results in Fig. 2 show that StringTie [12] can reliably profile a transcriptome even in the absence of an input guide annotation, while methods like Bambu [9] or FLAIR [8] demonstrate a substantial decrease in both sensitivity and precision of transcript identification as the percentage of origin transcripts in the input guide annotation is progressively reduced. Figure 2A shows that while Bambu outperforms StringTie and FLAIR in terms of average sensitivity when a substantial portion of the origin transcriptome is provided in the input, StringTie consistently outperforms the rest of the tools in precision across all percentages of origin transcripts kept in the input annotation. Bambu achieved highest F1 scores when the guides retained most of origin transcripts, but StringTie gradually surpassed others as guides became increasingly incomplete (also see Additional file 1: Table S3). This trend is consistent in Fig. 2B where IsoQuant is included in the comparison. When 100% or 50% of origin transcripts are provided in the guide annotations, IsoQuant and Bambu achieved higher sensitivities compared to the rest. However, as the guides become more incomplete (0% origin transcripts or de novo mode), StringTie showed substantially higher sensitivities and better F1 scores than the other tools (also see Additional file 1: Table S4). Note that, as expected, the performance of quantification-only tools also deteriorated rapidly as the guides became increasingly incomplete (see Additional file 1: Table S5 and Additional file 2: Fig. S2).

A strong resilience to varying degrees of incompleteness in the input transcriptome is critical, especially for studies involving poorly annotated organisms or in cases where the RNA-seq sample contains many novel isoforms. However, StringTie does not assign individual reads to the transcripts it assembles, making it difficult for the user to check the reliability of the isoforms it assembles using long reads. By introducing TranSigner, we aimed to also address this gap, in addition to improving transcript quantification accuracies. Note that, from this point onward, we describe TranSigner and Oarfish results with their coverage-related options enabled, as these tools demonstrated their best performances with these options active.

**Fig. 3:**
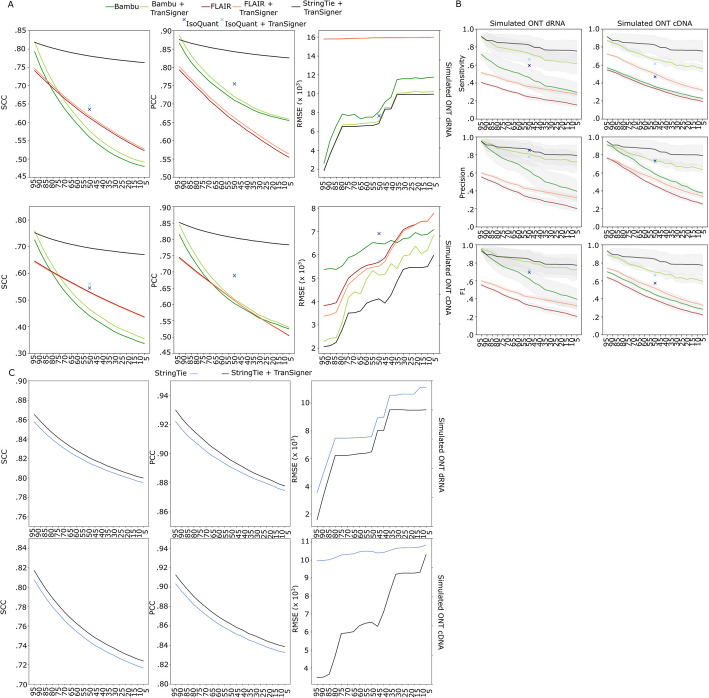
Impact of guide annotation completeness on transcript quantification and read assignment accuracies. **A** Average correlation coefficients and RMSE values for Bambu, Bambu + TranSigner, FLAIR, FLAIR + TranSigner, StringTie + TranSigner, IsoQuant, and IsoQuant + TranSigner comparing true and estimated read counts at varying percent guide annotations, computed using simulated ONT direct data (top) and ONT cDNA data (bottom). **B** Read-to-transcript assignment accuracies achieved by Bambu, Bambu + TranSigner, FLAIR, FLAIR + TranSigner, StringTie + TranSigner, IsoQuant, and IsoQuant + TranSigner on simulated ONT direct RNA data (left column) and ONT cDNA data (right column). Three metrics—sensitivity, precision, and recall—are displayed from top to bottom, with standard deviation (SD) values shown as shaded areas. Legend is shared between **A** and **B**. **C** Average correlation coefficients and RMSE values for StringTie and StringTie + TranSigner comparing true and estimated per-transcript coverages at varying percent guide annotations computed using simulated ONT data (ONT direct RNA results on the top and ONT cDNA results at the bottom)

Following our evaluation of transcriptome identification tools, we shifted our focus to assessing TranSigner’s performance through two carefully designed experiments using the sampled guides. The first experiment assessed TranSigner’s performance when paired with an existing tool capable of transcript identification. This scenario represents the typical use case for TranSigner, as RNA-seq data often includes novel transcripts. As shown by our previous results, including these novel transcripts in the transcriptome being quantified is important for its optimal performance. We first compared the performance of StringTie + TranSigner, Bambu + TranSigner, IsoQuant + TranSigner, and FLAIR + TranSigner to that of Bambu, IsoQuant, and FLAIR (see Fig. 3). As StringTie does not output read counts for its transcript abundance estimates, its performance was compared separately (see Fig. 3C). This comparison aimed to evaluate whether TranSigner could enhance transcript quantification performed by another tool and to identify which pairing delivers the most accurate estimates. Each combination or tool was evaluated for their quantification accuracies, as well as for their read assignment accuracies.

Average SCC and PCC values between the true and estimated read counts are shown in Fig. 3A (see Additional file 1: Table S6). Except for StringTie + TranSigner, all tools or tool combinations experienced a drastic drop in SCC and PCC values as the percentage of origin transcripts decreased. StringTie + TranSigner consistently produced the highest SCC values in both ONT direct RNA and cDNA benchmarks across varying levels of annotation incompleteness. We also observed that running TranSigner on transcripts identified by Bambu and FLAIR improved their correlation coefficients and reduced RMSE values. On direct RNA data, TranSigner lowered Bambu’s RMSE values to levels comparable to those obtained by StringTie + TranSigner. IsoQuant, as noted earlier, was only benchmarked with 50% complete guide annotations. While it achieved the second-best SCC and PCC values, it still lagged far behind the top contender. Furthermore, IsoQuant’s RMSE values were substantially higher on cDNA data compared to direct RNA data and were the highest RMSE among all tools. Running TranSigner on IsoQuant’s results significantly reduced these high RMSE values in simulated cDNA data.

To compare StringTie’s performance with that of StringTie + TranSigner, we post-processed TranSigner’s read-to-transcript assignments to generate read per base coverages, which are also output by StringTie (see Methods). As shown in Fig. 3C, StringTie + TranSigner, on average, achieved better read-per-base-coverage SCCs and PCCs than StringTie alone, while also substantially reducing RMSE values (see Additional file 1: Table S7). These results were consistent across both simulated ONT direct RNA and cDNA datasets, demonstrating that StringTie + TranSigner provided the most accurate transcript abundance estimates among all tools and tool combinations tested.

We next evaluated the read assignment accuracies of the same set of tools, excluding StringTie, which does not provide read assignments (see Fig. 3B). Bambu + TranSigner demonstrated the highest sensitivity and precision when the guides are nearly complete (i.e., 90 ~ 95% completeness), slightly outperforming StringTie + TranSigner in these cases. However, as the guides became less complete, StringTie + TranSigner consistently showed superior sensitivity, precision, and F1 values, aligning with the trends observed in Fig. 3A (Additional file 1: Table S8). Additionally, we observed that TranSigner can accurately assign reads to transcripts identified by FLAIR and Bambu, resulting in notable improvements across all metrics, while also increasing the percentage of total reads assigned (see Additional file 2: Fig. S3).

**Fig. 4:**
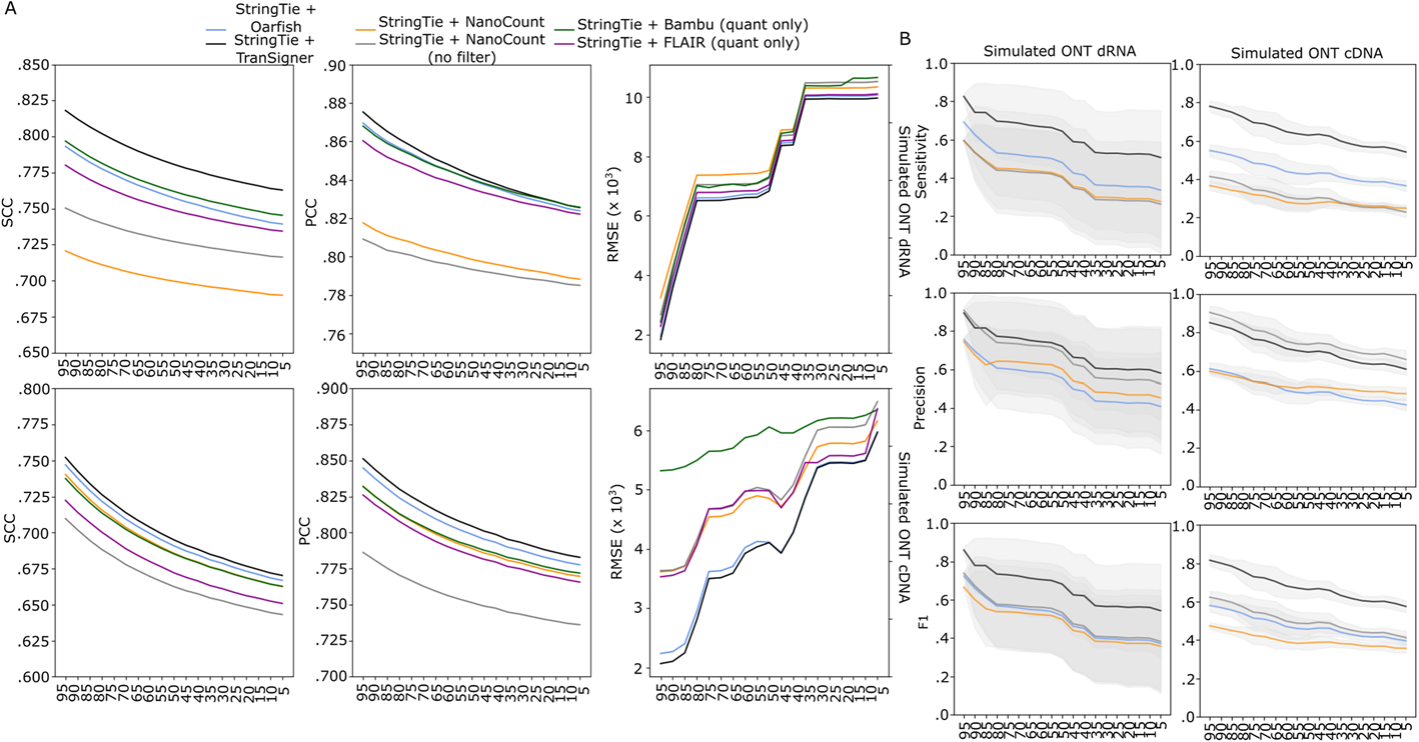
Abundance estimation and read-to-transcript assignment accuracy for StringTie + [TranSigner, Oarfish, NanoCount, NanoCount (no filter), Bambu (quant-only), FLAIR (quant-only)]. **A** Average correlation coefficients and RMSE values comparing true and estimated abundances computed at varying percent guide annotations computed using simulated ONT direct RNA (top) and ONT cDNA (bottom) data. **B** Read-to-transcript assignment accuracies on simulated ONT direct RNA (left column) and ONT cDNA (right column) data. Three metrics—sensitivity, precision, recall—are shown from top to bottom. Standard deviation (SD) values are shown as shaded areas. NanoCount was excluded from this evaluation as it does not output read assignments. Legend is shared between **A** and **B**

Our second experiment using the sampled guides aimed to determine whether pairing another quantification-only method with StringTie could outperform StringTie + TranSigner. Figure 4 shows the results of comparing StringTie + TranSigner to StringTie + NanoCount (with and without its filters), StringTie + Bambu (excluding its identification step), StringTie + FLAIR (excluding its identification step), and StringTie + Oarfish. As observed before in Fig. 3, StringTie + TranSigner outperformed the rest both in terms of the quality of transcript abundance estimates and read assignment accuracies (see Fig. 4 and Additional file 1: Tables S9 and S10).

### Real data performance

To evaluate the performance of TranSigner using experimental data, we utilized the ONT RNA-seq datasets provided by the Singapore Nanopore Expression Project (SG-NEx) [21], which include synthetic spike-in transcripts, known as sequins, with known annotation and concentrations. We selected 12 ONT direct RNA and cDNA samples from three different human cell lines: HCT116, K562, and MCF7 to assess how accurately quantification-only tools and transcript identification tools can quantify the sequins. For quantification-only tools, we chose TranSigner, Bambu with its identification feature disabled, and Oarfish—the top three performers in the quantification-only category based on our simulated data benchmarks—and ran them with the full SG-NEx-provided GRCh38 and sequin annotations. To evaluate how well the sequin transcripts can be recovered, we also ran Bambu and StringTie + TranSigner on the same samples but excluded the sequin annotations from the guides provided to them. For all tools evaluated, we computed both SCC and PCC between the ground truth and estimated CPM values (see Methods).

**Fig. 5:**
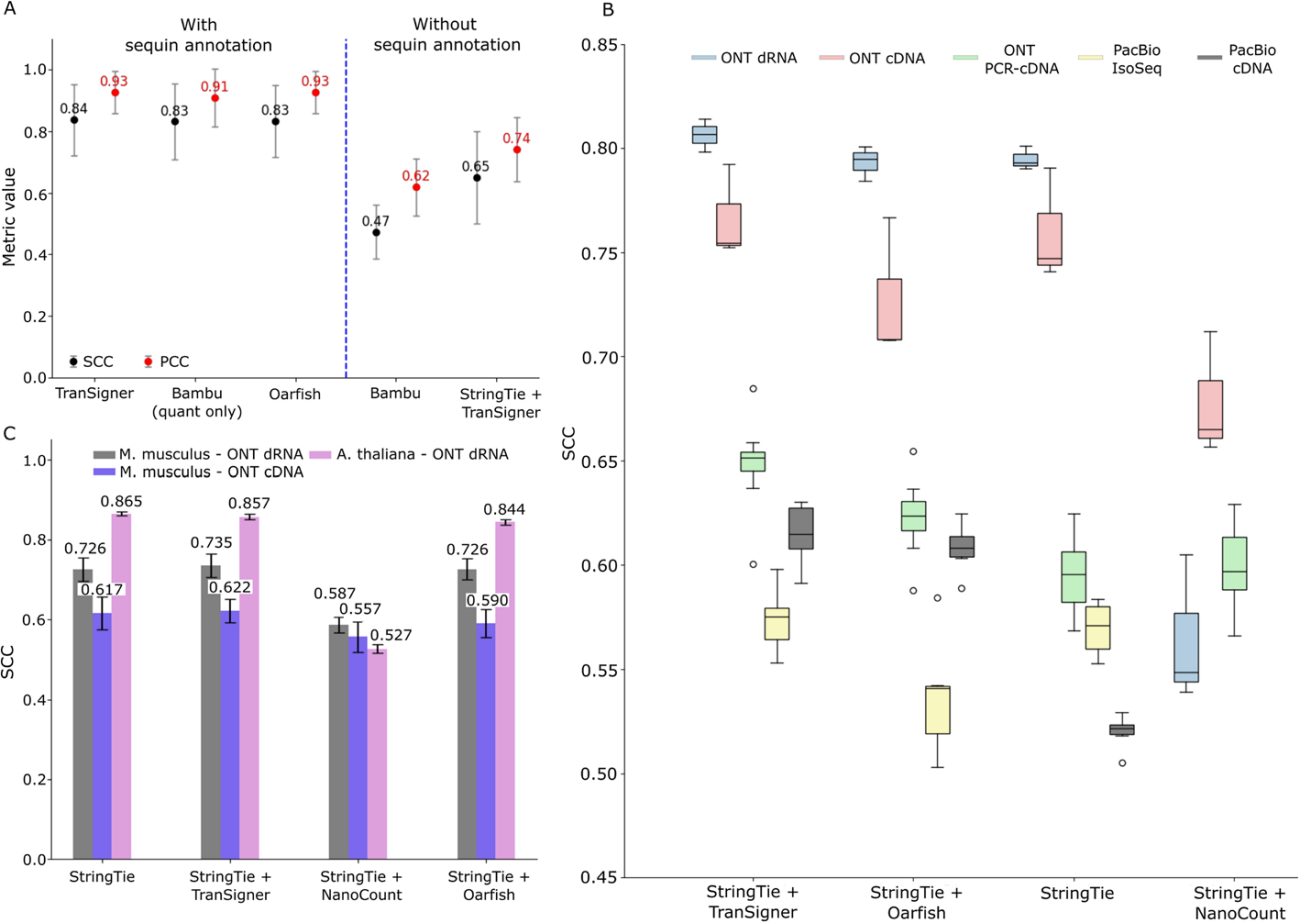
Real data transcript abundance estimation accuracy across multiple long-read quantification methods. **A** Correlation coefficients between estimated and expected sequin abundances. Quantification-only methods, TranSigner, Bambu (excluding its identification step), and Oarfish were evaluated with sequin annotations, while Bambu and StringTie + TranSigner were tested without sequin guidance. Error bars indicate standard deviation (SD) across 12 samples. **B** Box plots showing the distribution of SCC values, measuring the concordance between short- and long-read-derived transcript abundances for 27 human dataset pairs. TranSigner, Oarfish, and NanoCount were run on the StringTie assemblies of long-read samples, with StringTie’s initial estimates included for comparison. Five distinct read types are represented by different colors. **C** Bar plots displaying average SCC values between short- and long-read-based abundance estimates on StringTie’s long-read-derived assemblies. Different colors represent specific combinations of organisms and long-read data types. Error bars indicate standard deviation (SD) across the dataset pairs

Figure 5A shows the average SCC and PCC values achieved by the five tools evaluated. The three quantification-only tools achieved comparable SCC and PCC values. While TranSigner demonstrated the highest average SCC of 0.84, the margin compared to the other two tools was just 0.01, making the distinction negligible. Similarly, Bambu (quantification only) had a slightly lower average PCC value of 0.91 compared to 0.93 for TranSigner and Oarfish. A more notable gap in performance is observed between Bambu and StringTie + TranSigner. StringTie + TranSigner achieved a higher average SCC of 0.65 and PCC of 0.74 compared to Bambu (average SCC = 0.47; average PCC of 0.62). Although the limited number of sequins might cause these correlations to appear smaller or larger than they would with larger annotations, these results suggest that StringTie + TranSigner may be preferable in scenarios where numerous unannotated or novel isoforms are anticipated, while TranSigner is optimal when the reference is presumed to be nearly complete (also see Additional file 1: Table S11).

We also evaluated the correlation between short-read-based and long-read-based abundance estimates using publicly available paired short- and long-read datasets, sequenced from the same biological sample. In the results that follow, all short-read libraries were generated through poly-A selection and sequenced with Illumina sequencers, while the long reads were primarily generated using ONT direct RNA or cDNA sequencing protocols. Unlike the sequin samples or simulated long reads, the ground truth is unknown for these datasets, as we lack information on which transcripts are expressed and their relative abundances. However, it is generally assumed that short reads provide more accurate abundance estimates compared to long reads, as they are less error-prone and typically yield more reads. This assumption has been supported by prior literature, most notably in the conclusions from LRGASP’s extensive assessment of different combinations of RNA-seq protocols-platforms, including cDNA-Illumina data. Pardo-Palacios et al. employed a range of metrics, such as the cross variation of abundance estimates among replicates and SCC values observed in spiked-in and simulated data where the ground truth is known. Their analysis showed that cDNA-Illumina achieved top overall performance across all protocols and platforms, ranking among the best in nearly all evaluation metrics. For this reason, we treated short-read-derived abundance estimates as controls, a strategy also employed by the authors of Oarfish [14].

For this benchmark, we computed long read-based abundance estimates using three quantification-only tools: NanoCount, TranSigner, and Oarfish. All tools were provided with a StringTie-assembled transcriptome, which reflects a typical use case where users supply transcriptomes assembled from their samples of interest. We used each tool’s abundance estimates to conduct nonlinear correlation analyses between the short read-derived TPM estimates and long read-derived CPM. As previously done for benchmarking long-read quantification tools [13], we assumed that a higher correlation between long-read- and short-read-derived abundance estimates is indicative of a higher quantification accuracy. Since none of the three quantification-only tools outputs TPMs, we processed the read counts they provide to obtain counts per million (CPM) estimates, which are equivalent to TPMs in a long-read RNA-seq experiment where each read is considered to represent a transcript (see Methods for the read counts to CPM conversion equation). To obtain TPM estimates from short-read data, we used Salmon [22] with the Illumina datasets on the StringTie assemblies derived from long-read data (see Additional file 3: Note S1). Because transcripts with low abundances are prone to misassembly and are often excluded from downstream analyses, we only included in our results transcripts with TPM values greater than 1 as estimated by Salmon.

We first selected 27 paired short- and long-read datasets: 9 pairs from the SG-NEx datasets [21], 12 pairs from the Dong et al. datasets [23], and 6 pairs from the LRGASP datasets [24]. These datasets include long-read data generated using both ONT and PacBio platforms, with five different capture strategies: ONT direct RNA (3 pairs), ONT direct cDNA (3 pairs), ONT PCR-cDNA (9 pairs), PacBio cDNA (6 pairs), and PacBio IsoSeq (8 pairs) (see Additional file 1: Table S12 for detailed cell line information).

As illustrated in Fig. 5B, both StringTie + TranSigner and StringTie + Oarfish achieved higher median SCC values compared to StringTie alone. StringTie + Oarfish showed improvements over StringTie with ONT direct RNA, ONT PCR-cDNA, and PacBio cDNA data, and declines with ONT direct cDNA and PacBio IsoSeq data. StringTie + NanoCount resulted in substantial declines in SCC values for all types of ONT data, except for ONT PCR-cDNA. StringTie + TranSigner was the only pair that did not cause a reduction in correlation values across all data types, including those sequenced on PacBio platforms, and achieved the highest degree of improvement across all types of long-read data (see Additional file 1: Table S12). Note that NanoCount was not evaluated on PacBio data, as it was designed specifically to work with ONT data only.

Finally, we expanded our benchmark to include paired short- and long-read datasets from two well-studied species: *Arabidopsis thaliana* [25] and *Mus musculus* [26]. To investigate how quantification accuracies vary at different levels of expression, we evaluated the performance of StringTie and combinations of StringTie with TranSigner, NanoCount, and Oarfish. For this experiment, we selected eight *M. musculus* pairs (four ONT direct RNA, four ONT cDNA) and three *A. thaliana* pairs (all ONT direct RNA). We measured the concordance between the short- and long-read-based abundance estimates on unguided StringTie assemblies, consistent with the previous analysis. As illustrated in Fig. 5C, TranSigner was able to improve the concordance (i.e., SCC values) observed *in M. musculus* ONT direct RNA and cDNA data. Surprisingly, both NanoCount and Oarfish failed to show improvement in SCC compared to the StringTie baseline estimates. Moreover, all three quantification-only tools struggled to improve the SCC values in *A. thaliana* data, an issue that remains unresolved (see Additional file 1: Table S13).

## Discussion

In benchmarks on simulated ONT data and experimental data from both ONT and PacBio platforms, TranSigner achieved state-of-the-art quantification and read assignment accuracies. However, it is important to acknowledge the limitations of conclusions derived from tests performed on simulated data. Simulated data often fails to capture the characteristics of real data (e.g., 3′ end coverage bias) and can introduce its own set of technical artifacts and biases specific to a simulator. For this reason, the choice of simulator can lead to different observations and conclusions, and further evaluation of TranSigner’s performance should be carried out using additional experimental data. Our read assignment evaluations were limited to simulated reads, as this information is practically unknown in experimental datasets. Unlike transcript abundances, which can be approximated using the concordance between short- and long-read-based estimates or measured using spiked-in data, there is currently no established method for inferring the ground truth read-to-transcript assignments in experimental data. We also recognize that our simulation was limited to ONT data and could be expanded in the future to include PacBio data, allowing for a more thorough evaluation of TranSigner’s read assignment accuracy on PacBio-generated reads. Further development of methods to address this gap are necessary to add confidence in the performance of TranSigner and other tools designed for read-to-transcript assignments.

Furthermore, note that our assessment of abundance estimates focused on correlation with, and deviation from, ground truth read counts, which only partially captures the impact of FPs—those attributed to transcripts not present in the ground truth and corresponding to unexpressed transcripts in a real RNA-seq sample. We observed that FP read counts are typically low in magnitude (less than 1), often randomly distributed, and comparably prevalent across all tools evaluated. Including these counts in correlation analyses can distort the results, as false positive transcripts vastly outnumber ground truth transcripts, and each is weighted equally in the calculation. However, when we recomputed all abundance evaluation metrics after excluding all predicted read counts below 1 (see Additional file 1: Table S14), the relative performance of all benchmarked tools remained unchanged. We recommend that users filter out transcripts with read count estimates below 1 and disregard all read-to-transcript assignments involving these likely false positive transcripts.

## Conclusions

Assigning long RNA-seq reads to transcripts is a challenging task that involves the effective resolution of multi-mapping reads. Recent studies have unveiled the growing complexity of eukaryotic transcriptomes, revealing numerous isoforms across gene loci. The introduction of long-read RNA-seq technologies promises to uncover even more novel isoforms, as reads produced by these methodologies can capture full-length transcripts, overcoming the limitations of short reads. Although long reads cover transcripts at greater lengths, technical artifacts such as base calling errors and end truncations prevent these reads from being accurately mapped to their origins. With TranSigner, we have developed several strategies to address this challenge, facilitating the correct assignment of reads that ambiguously map to multiple isoforms.

A central focus of our work was to design TranSigner to complement another method capable of transcriptome assembly. As gene annotation is still an unresolved issue, determining the accuracy and completeness of a profiled transcriptome remains difficult. Users often struggle to select the appropriate reference for their analyses, leading to unpredictable impacts on their results. In our study, we observed a significant drop in assembly quality when less complete guides were provided. This suggests that tools heavily reliant on high-quality reference annotations may struggle in real-world scenarios where many novel isoforms are expected. By introducing a standalone tool for read-to-transcript assignments, we have made these assignments easier to obtain regardless of the input transcriptome. Integrating this step into long-read RNA-seq data processing pipelines will improve the accuracy of transcriptomes identified using long reads by allowing users to inspect the quality of the reads supporting the transcripts and filter out less-supported ones. This, in turn, will lead to more accurate abundance estimates, as our results demonstrate the significant impact of assembly accuracy on the correct identification of transcript abundances.

## Methods

**Fig. 6:**
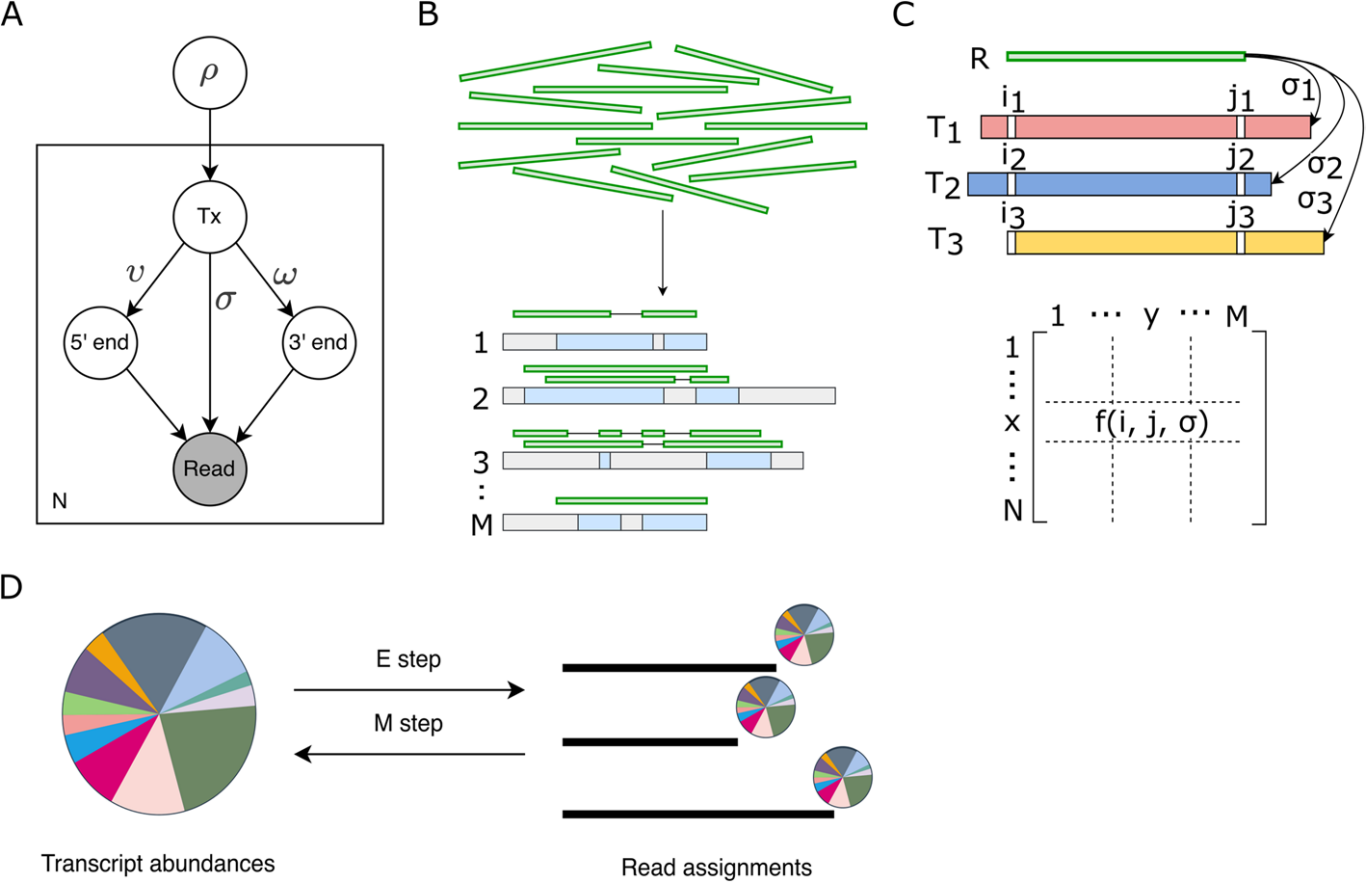
**A** Graphical representation of TranSigner’s long-read RNA-seq model. Empty circles denote latent variables and the shaded circle represents the observed variable. Parameters $\upsilon$ and $\omega$ approximate the likelihood of the specific 5′ and 3′ end positions of the read on the transcript, while parameter $\sigma$ models the likelihood of observing a specific read sequence given a transcript and the read’s end positions. $N$ represents the total number of reads generated in a single long-read RNA-seq experiment. **B**–**D** Schematic showing TranSigner’s workflow consisting of three modules. **B** In the align module, long RNA-seq reads are mapped to an input transcriptome containing transcript sequences in non-spliced mode. **C** In the prefilter module, compatibility scores are precomputed using alignment positions and scores, and highly improbable alignments can be optionally removed. **D** In the EM module, read fractions are assigned to transcripts and transcript abundances are updated iteratively until convergence

We describe the long-read RNA-seq process using a generative model (Fig. 6A). The conceptualization of RNA-seq as a generative process in which reads are sampled from a pool of transcripts has already been used in models for short-read quantification. We adopted the general framework proposed by others [3, 27] but introduced necessary modifications to tailor the model to long-read data. Given a read, we assume that three unobserved events in the RNA-seq experiment determine a read’s sequence: (1) the transcript from which that read was sequenced, (2) the 5′ end and (3) 3′ end positions on the transcript. Our model, thus, associates each observed read with three latent variables: the transcript ($T$) from which the read was generated, its 5′ end position ($S$), and 3′ end position ($E$) in $T$.

Existing EM-based RNA-seq quantification methods focus on obtaining the maximum likelihood (ML) estimate of relative transcript abundances ($\rho$), the primary parameter used to describe RNA-seq data [3, 14, 27]. The EM algorithm iteratively updates both the expected values of latent variables and model parameter estimates. In this context, the latent variables correspond to read-to-transcript assignments ($\alpha$). Previously, the expected values for $\alpha$ have primarily been used to compute ML estimates for $\rho$. However, our approach emphasizes the accuracy of these values and their interpretations as either fractional or discrete assignments of reads to transcripts, shaping the design of the TranSigner algorithm. Methods developed for different purposes (e.g., quantification versus read assignments) evaluate their estimates primarily for quantification accuracies. However, TranSigner was assessed for both read assignment and quantification performance. To address the unique challenges of long RNA-seq read assignment, we adapted the standard EM algorithm by introducing critical modifications, such as position-specific weights and the drop/push features described below. Most of these innovations apply to the E-step update rule, which determines how read fractions are distributed across transcripts, given a fixed $\rho$.

Given a set of transcripts $T=\left\{t\right\}$ where $\left|T\right|=M$, the complete data likelihood function of our RNA-seq model is:

1 $$\mathcal{L}\left(\rho \right)=\prod_{r\in R}\sum_{t\in T}\begin{array}{c}{\rm P}\left(r\in t|\rho \right){\rm P}\left({s}_{rt}|r\in t\right)\\ {\rm P}\left({e}_{rt}|r\in t\right){\rm P}\left(r|r\in t,{s}_{rt},{e}_{rt}\right)\end{array}$$

where $$\rho ={\{{\rho }_{t}\}}_{t\in T}$$ with $${\sum }_{t\in T}{\rho }_{t}=1$$, $$R$$ is the set of mapped reads defined as $$R=\{r\}$$ with the cardinality of $$N$$, $${s}_{rt}$$ and $${e}_{rt}$$ are the 3′ and 5′ end positions of a read $$r$$ in a transcript $$t$$, and $$r\in t$$ indicates that $$r$$ comes from $$t$$. Note $$\text{\rm P}(r\in t|\rho )={\rho }_{t}$$, since in an RNA-seq experiment the probability of selecting a transcript *t* to sequence depends on its relative abundance. We will approximate the 5′ end ($${s}_{rt})$$ and 3′ end ($${e}_{rt}$$) positions of a read in a transcript as the positions where the read alignment starts and ends on that transcript, respectively. The relationship between this likelihood function and read assignment estimates is easier to understand when Eq. 1 is rewritten as:

2 $$\mathcal{L}\left(\rho \right)=\prod_{r\in R}\text{\rm P}(r|\rho )=\prod_{r\in R}\sum_{t\in T}\text{\rm P}(r|r\in t,\rho )=\prod_{r\in R}\sum_{t\in T}{\alpha }_{rt}$$

where ${\alpha }_{rt}$ is the relative fraction of read $r$ assigned to transcript $t$ given some $\rho$. $\text{\rm P}(r|\rho )$ can also be written as a sum of conditional probabilities $\text{\rm P}(r|r\in t,\rho )$, which represents the likelihood of $r$ given it comes from $t$. This conditional probability is also easily interpretable as the fraction of $r$ that ought to be assigned to $t$, implying that a lower $\text{\rm P}(r|r\in t,\rho )$ corresponds to a smaller ${\alpha }_{rt}$. Moreover, optimizing $\mathcal{L}$ involves driving $P(r|\rho )$ to the maximum possible value in a probability distribution (i.e., 1), which is also equal to the sum of relative fractions of a read’s assignments to the set of transcripts (i.e., $\sum_{t\in T}{\alpha }_{rt}=1$).

### Optional alignment filters

Different long-read RNA-seq technologies show various biases towards the ends of the transcripts [21, 28–30]. Nonetheless, long reads are more likely to cover all bases of a transcript, compared to short reads, which are generated from fragments of the transcript. The likelihood of a read’s end position should decrease as its distance from the transcript end increases. We model this expectation using two indicator variables—$\upsilon$ and $\omega$ for the 5′ and 3′ transcript ends, respectively—to control how far apart the ends of a read can be from the ends of a transcript. For an alignment between a read $r$ and a transcript $t$, we will refer to the distances between the alignment ends and transcript ends as “end distances” and denote them as ${\delta }_{s}^{rt}$ and ${\delta }_{e}^{rt}$ for the 5′ and 3′ ends, respectively. Then, we define $\upsilon$ and $\omega$ as:

3 $$\begin{array}{c}\mathrm P\left(S_{\mathit r\mathit t}\mathit=i\left|\mathit r\mathit\in\mathit t\right.\right)\mathit\approx{\mathrm v}_{\mathrm{rt}}\mathit=\mathit1\mathit\;if\left|\mathrm\delta_{\mathrm s}^{\mathrm{rt}\mathit'}\right.\mathit-\delta_{\mathit s}^{\mathit r\mathit t}\left|\mathit\leq\beta_{\mathit s}\mathit,\mathit\;0\mathit\;\mathrm o\mathit.\mathrm w\mathit.\right.\\\mathrm P\left(S_{rt}\mathit=j\left|r\mathit\in t\right.\right)\approx{\mathrm\omega}_{\mathrm{rt}}=\left|1\;\mathrm{if}\left|\delta_{\mathit s}^{\mathit r\mathit t\mathit'}\mathit-\delta_{\mathit s}^{\mathit r\mathit t}\left|\mathit\leq\right.\right.\;\beta_s\mathit,\mathit\;0\;\mathrm o.\mathrm w.\right.\\\end{array}$$

where ${\delta }_{s}^{{rt}{\prime}}$ and ${\delta }_{e}^{rt{\prime}}$ represent the end distances observed in the primary alignment of the read $r$, determined by minimap2 based on alignment scores.

Here, $t{\prime}$ represents the transcript to which read $r$ aligns in its primary alignment, which might not be the same as transcript $t$. Since alignment positions are indexed from the 5′ to 3′ direction on transcript $t$, end distances are computed as ${\delta }_{s}^{rt}=i$ and ${\delta }_{e}^{rt}=\left|t\right|-j$ where $|t|$ is the length of transcript $t$ and $i$ and $j$ are the alignment start and end positions. Parameter $\beta$ represents the tolerance threshold on how much greater the end distances can be compared to the primary alignment’s end distances for a given read $r$. This relative thresholding on end distances ($\delta$) ensures that each read is compatible with at least one transcript (i.e., $t{\prime}$) after this filtering step since the primary alignment will always be considered “good,” which would not be true if a constant threshold was uniformly applied for all reads. When either $\upsilon$ or $\omega$ is set to 0, $\text{\rm P}(r|r\in t)$ in Eq. 2 is also set to 0, and no fraction of $r$ is assigned to $t$, guaranteeing that the corresponding $(r, t)$ pair will be considered entirely incompatible, filtering it out from any downstream analysis.

Moreover, the parameters for the 3′ end are treated separately from those for the 5′ end because sequencing behaves differently at these ends. For example, there is a stronger coverage bias towards the 3′ end when nanopore-based direct RNA sequencing protocols are employed [21, 28–30]. We set the $\beta$ parameter values based on both prior knowledge and a grid search (see Additional file 3: Note S2). For the ONT direct RNA data, the recommended values are ${\beta }_{s}=-800$ and ${\beta }_{e}=-\infty$ (i.e., 3′ end filter deactivated), while for ONT cDNA and PacBio data, they are ${\beta }_{s}=-600$ and ${\beta }_{e}=-500$ for ONT cDNA and PacBio data. During the grid search, we observed that filtering out alignments based on these distances did not significantly improve the correlation between estimated and expected transcript abundances (see Additional file 3: Note S2). As a result, we made these filter completely optional, as their optimal thresholds likely vary depending on the library protocol and sequencing platform, making one-size-fits-all recommendations impractical. They are available for users who wish to exclude alignments based on prior knowledge of where alignments should start and end on transcripts. We also observed that the 5′ end distances are only weakly correlated with transcript lengths (see Additional file 2: Fig. S4).

### Position-specific weights

When the *--psw* flag is activated, TranSigner computes the likelihood that a read $r$ covers base positions ${s}_{rt}$ through ${e}_{rt}$, given $r\in t$, corresponding to $\text{\rm P}\left({s}_{rt}=i, {e}_{rt}=j|r\in t\right)$, equivalent to the product term $\text{P(}{s}_{rt}=i\left|r\in t\right)\bullet \text{P(}{e}_{rt}=j|r\in t)$ in Eq. 1 assuming $\text{P(}{s}_{rt}=i)$ and $\text{P(}{e}_{rt}=j)$ are conditionally independent given $r\in t$. Our approach was inspired by Jousheghanis et al., who proposed increasing this conditional likelihood for sparsely covered base positions while decreasing it for overly covered regions. The authors suggest modeling this probability using a binned approach, where read counts are tallied within transcript sections and processed into a probability distribution that assigns higher values to regions with relatively low coverage. Instead of the binned approach, we present the following formulation:

4 $$\mathrm P\left(e_{rt}=i,s_{rt}=j\left|r\in t\right.\right)\approx{\textstyle\sum_{k=i}^j}\;\eta_k^"=f_\eta\left(r,t\right)$$

where ${\eta }_{k}^{{\prime}{\prime}}={\eta }_{k}{\prime}/{\sum }_{z=1}^{l(t)}{\eta }_{k}{\prime}$, ${\eta }_{k}{\prime}=|{\text{min}}_{z=1}^{l\left(t\right)}{\eta }_{z}|$, and ${\eta }_{k}=\left({c}^{t}-{c}_{k}^{t}\right)$. ${c}^{t}$ and ${c}_{k}^{t}$ are per-transcript coverage estimate for transcript $t$ and per-base coverage estimate for base position $k$ of $t$, respectively. More specifically, ${c}_{k}^{t}$ is computed by tallying the number of reads aligned to base position $k$ of $t$, while ${c}^{t}$ is obtained by dividing the total number of read bases aligned to $t$—equivalent to the sum of distances between alignment start and end positions on $t$ (i.e., aligned transcript region lengths)—by its length. ${\eta }_{k}^{{\prime}{\prime}}$ are normalized and zero-shifted differences, calculated by subtracting the per-base coverage estimates from the corresponding transcript’s coverage. The intended effect of this model is to assign higher values to base positions with coverage values much lower than the transcript-wide coverage, compared to bases with higher coverages. We refer to these ${\eta }_{k}^{{\prime}{\prime}}$ values as position-specific weights rather than conditional probabilities, as they do not follow the basic requirements of a probability distribution.

Unlike the coverage model proposed by Jousheghanis et al., our approach does not require post-processing binned read counts into probabilities. Instead, simple calculations of per-base and per-transcript coverages are sufficient to prioritize alignments that overlap sparsely covered base positions within a transcript.

### Alignment scores

The probability of observing a read $$r$$ given all the latent variables is modeled using an exponential decay function with a fixed parameter $$\lambda$$ (default value = 5) representing decay rates. Given an alignment score between read $$r$$ and transcript $$t$$ (denoted by $${x}_{rt})$$ as:

5 $${P}\left(r|r\in t,{s}_{rt}=i,{e}_{rt}=j\right)\approx {\sigma }_{rt}={e}^{(-1*\left(\underset{k\in T}{\text{max}}{x}_{rk}-{x}_{rt}\right)/\lambda )}$$

This decay function models an exponentially decreasing likelihood of read assignment as the alignment score’s distance from the maximum increases. Previous studies have shown that alignment score distributions often exhibit exponential tail behavior and have been approximated using extreme-value distributions [31] for significance estimation or Boltzmann distributions for posterior probabilities, supporting our use of an exponentially decaying function [32–35]. Note that if multiple alignments exist between read $r$ and transcript $t$, we only retain the alignment with the maximum score. Using the above definitions, we can redefine the likelihood function as:

6 $$\mathcal{L}\left(\rho \right)=\prod_{r\in R}\sum_{t\in {T}_{r}}{\rho }_{t}{\upsilon }_{rt}{\omega }_{rt}{f}_{\eta }(r,t){\sigma }_{rt}$$

where ${T}_{r}$ is the set of transcripts aligned to read $r$, with ${\upsilon }_{rt}$, ${\omega }_{rt}$, and ${\sigma }_{rt}$ set to zero for any unaligned pair of read $r$ and transcript $t$. By default (i.e., drop mode activated), ${f}_{\eta }(r,t)$ is equal to 1. However, if the psw mode is activated, it is calculated as described in Eq. 6.

By combining Eqs. 2 and 7, we obtain that:

7 $${\rho }_{t}{\upsilon }_{rt}{\omega }_{rt}{f}_{\eta }(r,t){\sigma }_{rt}={\alpha }_{rt}$$

which shows how ${\alpha }_{rt}$ can be computed from the alignments between reads and transcripts, assuming that the relative transcript abundances are given. In the psw mode, we observed edge cases where ${\sum }_{t\in {T}_{r}}{f}_{\eta }(r,t){\sigma }_{rt}=0$ (approximately 0.32% on average across our experimental data sets), which most likely occur when a read overlaps a low abundance transcript, as ${f}_{\eta }(r,t)$ approaches zero when the per-base coverages for aligned positions becomes very small. When $\sum_{t\in {T}_{r}}{f}_{\eta }(r,t){\sigma }_{rt}=0$, some reads remain unassigned. To address this, TranSigner identifies such cases and avoids multiplication by ${f}_{\eta }(r,t)$, reverting to the drop model for this specific case, and setting ${f}_{\eta }(r,t)$ equal to 1. This indicates a potential limitation of coverage-based approaches when insufficient coverage data is available for relatively lowly expressed transcripts.

### Alignment

We used minimap2 (v2.26) with parameter -N 181 to align the long reads to the set of input transcripts [16, 17]. By default, minimap2 limits the maximum number of secondary alignments to 5. We observed that the number of true positives (correct read to transcript alignments) increases when we retain more secondary alignments, so we set -N to 181, the highest number of transcripts in a single gene locus according to the GRCh38 RefSeq annotation (release 110), assuming this is the maximum number of secondary alignments a read can have. This strategy provides rough, preliminary estimates on the compatibility between reads and transcripts, without excluding any read and transcript pair for having suboptimal alignment scores. The user can freely adjust this parameter by specifying it in TranSigner’s input, which will then pass it to minimap2.

### Alignment-guided expectation–maximization algorithm (AG-EM)

Our primary goal is to accurately assign reads to their respective transcript origins. We previously introduced $\alpha$ as a variable representing read-to-transcript assignments and established that the distribution over $\alpha$ is equivalent to that over the latent variables of our long-read RNA-seq model (Fig. 6B–D and Eqs. 1, 2, 4). An expectation–maximization (EM) algorithm estimates the maximum likelihood (ML) of model parameters by iteratively updating the expected values of the latent variables. Hence, TranSigner employs EM to obtain the most probable—in the sense that the complete data likelihood is maximized—distribution over $\alpha$ and presents the corresponding expected values as read-to-transcript assignments. It also outputs the ML estimates on $\rho$.

#### Update rules

The EM algorithm consists of alternating expectation (E) and maximization (M) steps, repeated until convergence. During the E step, the expected values for ${\alpha }_{rt}^{(n)}$—at some iteration $n$—are computed as follows:

8 $${\alpha }_{rt}^{(n)}=\frac{{\rho }_{t}^{(n)}{\upsilon }_{rt}{\omega }_{rt}{\sigma }_{rt}}{\sum_{{t}{\prime}\in {T}_{r}}{\rho }_{{t}{\prime}}^{(n)}{\upsilon }_{r{t}{\prime}}{\omega }_{r{t}{\prime}}{\sigma }_{r{t}{\prime}}}$$

where ${\alpha =\{{\alpha }_{rt}\}}_{r,t\in A}$ and $A$ is the set of alignments between all reads and transcripts. In the following M step, then, the fragments of reads assigned to each transcript are summed up and then normalized by the total number of transcripts to get the relative transcript abundances, expressed as:

9 $${\rho }_{t}=\frac{\sum_{r\in {R}_{t}}{\alpha }_{rt}}{\sum_{{r}{\prime},{t}{\prime}\in A}{\alpha }_{{r}{\prime}{t}{\prime}}}$$

where ${R}_{t}$ is the set of reads aligned to transcript $t$. The denominator is constant across iterations and is equivalent to the total number of reads in a long-read RNA-seq experiment where each read represents a transcript, so we precompute this value before EM.

#### Initialization

Before the EM iterations, the relative transcript abundances ($\rho$) are initialized to the uniform distribution:

$${\rho }_{t}=\frac{1}{|{T}_{A}|}$$

where ${T}_{A}$ is the set of transcripts with at least one alignment to a read in $R$. Additionally, the values for $\upsilon$, $\omega$, and $\sigma$ do not change during iterations, so we precompute their values and store them separately in a matrix $X$ of dimensions $N$ rows and $M$ columns. For simplicity, we will refer to $X$ as the compatibility score matrix. The computation specified in Eq. 10 is further simplified as:

10 $${\alpha }_{rt}^{(n)}=\frac{{\rho }_{t}^{(n)}{X}_{rt}}{\sum_{{t}{\prime}\in {T}_{r}}{\rho }_{{t}{\prime}}^{(n)}{X}_{r{t}{\prime}}}$$

The pre-computation step involves a single scan over the alignment results, extracting values such as the alignment scores and alignment start/end positions, and then applying the definitions provided in Eqs. 4 through 6.

#### Optimization

Once $X$ is precomputed and $\rho$ is initialized, EM iterations are repeated until convergence, i.e., until the total sum of changes in the read counts is less than a predefined threshold, by default set at 10. The user can adjust this threshold to increase the accuracy of the ML estimates at the expense of speed.

The novelty of our method comes from guiding the EM algorithm with the priors extracted from the alignment results, as detailed in the E-step update rule shown in Eq. 12. To further amplify the impact of these priors, we implemented an algorithm called the *drop*. The *drop* algorithm (see Additional file 2: Fig. S5) sets ${X}_{rt}=0$ if the fraction of read $r$ that is assigned to transcript $t$ (i.e., ${\alpha }_{rt}$) gets below a threshold, $\tau \in [\text{0,1}]$. This effectively drops the compatibility relationship between read $r$ and transcript $t$ and ensures that no fraction of $r$ gets assigned to $t$ in any iterations following the drop, as ${\alpha }_{rt}$ will always be 0 since its computation involves multiplication by ${X}_{rt}$ (Eq. 12). After the drop, another E-step is performed with the updated $X$ scores to recompute the new ${\alpha }_{rt}$ values. The $\tau$ value depends on the read *r* considered, and by default:

11 $${\tau }_{r}=\frac{1}{|{T}_{r}|}$$

where ${T}_{r}$ is the set of transcripts that are compatible with $r$. The *drop* algorithm is called only right after the first E-step calculation, and its purpose is to discard minimap2 alignments that are not robust. The drop algorithm offers the potential to achieve a higher optimum compared to a naïve EM algorithm [27], which relies solely on the relative transcript abundances ($\rho$) in its E-step update. We also allow users to increase this threshold (i.e., make it stricter) using the *-df* parameter that will increment ${\tau }_{r}$ by a fraction of its own value as follows:

12 $${\tau }_{r}{\prime}={\tau }_{r}+({\tau }_{r}*f)$$

where $f$ is a fractional value within the range [0, 1]. By default, TranSigner employs this *drop* algorithm with $f$ set to 0.1.

#### Read assignment

We can use the $\alpha$ values estimated by the EM algorithm to infer read assignments to transcripts. Raw $\alpha$ values represent fractional read assignments, where a single read may be distributed among multiple transcripts. These assignments might be challenging to interpret, as we assume each read to originate from a single transcript. To increase the interpretability and usability of the $\alpha$ values, we implemented the *push* algorithm (see Additional file 2: Fig. S6). This algorithm processes raw $\alpha$ values, converting them into hard 1-to-1 assignments where each read is assigned to exactly one transcript.

Given a read *r*, the *push* algorithm iterates through all potential assignments (i.e., candidate transcripts) and collects the read fractions assigned to each. These read fractions naturally form a weighted discrete distribution, where the event of assigning $r$ to the transcript $t$ is attributed to a weight equivalent to the read fractions computed at the end of the EM iterations. The algorithm then samples an assignment from this distribution to establish a hard assignment for $r$. After repeating this step for all reads, the relative transcript abundances are recomputed based on the resulting hard assignments. These new $\alpha$ and $\rho$ values may deviate from their EM-derived ML estimates, potentially resulting in reduced accuracy.

Obtaining hard assignments can be challenging, as mistakes result in harsher penalties in terms of assignment accuracy and abundance estimates. We can measure the effectiveness of a specific hard assignment strategy based on its impact on the quality of read assignment and/or abundance estimates. Using simulated ONT direct RNA and cDNA data, we tested the impact of two hard assignment strategies: (1) assigning reads to the transcript with the highest fraction allocated by EM and (2) sampling assignments from a weighted discrete distribution defined by the read fractions computed during EM. We observed that the latter sampling strategy (2), employed by our *push* algorithm, incurred significantly smaller deterioration in SCC and PCC values, and almost no increase in RMSE (i.e., maximum of ≈3) for both ONT direct RNA and cDNA data, demonstrating its effectiveness in obtaining hard assignments. In contrast, the naïve strategy resulted in approximately 0.02 ~ 0.06 average drops in SCC and PCC values for simulated ONT data, numbers significantly greater than the average drops of 0.002 ~ 0.004 observed when the sampling strategy is employed (also see Additional file 1: Table S15). The maximum RMSE increase was ≈6 before, almost as twice as large as that observed for the sampling strategy. While the current implementation of the drop algorithm results in only a negligible decrease in accuracy, sampling from any distribution inherently introduces stochasticity. This randomness carries the risk of selecting incorrect assignments purely by chance. Future approaches, such as bootstrapping, ensemble methods, and Monte Carlo techniques, could mitigate this issue and further improve the quality of hard assignments that TranSigner generates.

### Implementation

TranSigner requires two inputs: a GTF file containing a reference gene annotation of the target transcriptome and a FASTQ file containing long RNA-seq reads. The reference annotation can be obtained from public sources such as RefSeq [36], GENCODE [37], or CHESS [20], or it can be derived from transcriptome assemblies produced by programs like StringTie. The latter annotations have the advantage of including novel isoforms while restricting the annotated transcripts to only those found to be expressed in the analyzed sample.

As illustrated in Fig. 6, TranSigner consists of three modules: align, prefilter, and em. In the align module, input long reads are aligned to the target transcriptome using minimap2. The resulting alignment file becomes the input for the next module. Next, in the prefilter module, TranSigner extracts features such as the 3′ and 5′ end alignment positions and the ms alignment scores computed by minimap2. These features are used to compute the compatibility score matrix between transcripts and reads, as well as an index of the IDs of the transcripts found to be compatible with reads in the align module, which represent a subset of the target transcriptome.

Finally, the EM module takes as inputs the compatibility score matrix and the target transcriptome index from the prefilter module. It estimates the transcript abundances using an expectation–maximization (EM) algorithm. The EM algorithm converges when the total change in read counts is less than a specified threshold, by default set to 10. We observed that TranSigner’s estimates improve with additional iterations. Considering the trade-off between speed and accuracy, we set our convergence threshold to allow for EM iterations in the hundreds rather than thousands, as improvements diminish with increasing iterations (see Additional file 2: Fig. S7). The *drop* algorithm is implemented as a component of this module. It allows users to remove low-compatibility relations between reads and transcripts immediately after the first E-step update. Read-to-transcript assignments (i.e., $\alpha$ estimates) and transcript abundances (i.e., $\rho$ estimates) are outputted as TSV files at the end of the EM module. Users also have the option to further process the assignments and output hard 1-to-1 assignments between reads and transcripts for increased interpretability by specifying the *--push* flag.

### Simulated data

Three sets of Oxford Nanopore Technologies (ONT) direct RNA reads and two sets of ONT cDNA reads were simulated using NanoSim [15]. We supplied the NA12878 direct RNA and cDNA reads from Workman et al. to NanoSim’s read characterization module to first construct two separate read profiles, one for generating direct RNA and the other for generating cDNA reads [38]. We then estimated the transcript abundances of the direct RNA and cDNA samples by aligning each sample to the GRCh38 genome using minimap2 and providing the alignment results to Salmon [22] in its alignment-based mode. Expression levels were derived from protein-coding and long non-coding transcripts located on the main chromosomes (i.e., chromosomes 1–22, X, and Y) of the GRCh38 RefSeq annotation (release 110) (see Availability of data and materials). For each direct RNA read set, we generated ~ 14 million ONT direct RNA reads, and ~ 25 million for each cDNA read set (see Additional file 3: Note S3). Using the known origins of simulated reads, we computed ground-truth read counts by counting the number of reads simulated from each transcript. Per-transcript coverage was calculated by summing the lengths of all reads simulated from a given transcript and dividing by its length.

For evaluation with simulated data, each tool was provided with the same RefSeq annotation used to quantify the Workman et al. ONT samples. Specifically, we used the RefSeq protein-coding and lncRNA transcripts annotated on the main chromosomes of GRCh38 as the target transcriptome for benchmarking tools on the simulated data. This reference annotation includes all transcripts that are expected to be captured in a poly(A)-selected RNA-seq sample. See Additional file 3: Notes S4 and S5 for the list of commands used to benchmark different tools capable of long-read RNA-seq quantification.

### Spiked-in data

We used an ONT direct-RNA dataset, which was released as part of the Singapore Nanopore Expression Project (SG-NEx) [21]. This dataset was sequenced from three different human cell lines, HCT116, K562, and MCF7, and includes synthetic sequencing spike-in RNAs, also known as sequin. When evaluating the performance of tools that rely on genomic alignments (e.g., StringTie, Bambu), we ran minimap2 in splice mode (i.e., *minimap2 -ax splice*) to map the spiked-in data to the custom SG-NEx-provided reference genome, which includes an in silico chromosome on which sequins are defined. For quantification-only methods that rely on transcriptomic alignments (e.g., TranSigner, Oarfish), we ran minimap2 in non-spliced ONT mode (i.e., *minimap2 -ax map-ont*) to map reads to sequin and GRCh38 transcript sequences extracted from the SG-NEx-provided annotation. We also obtained the sequin transcripts’ raw abundances and their sample-wise spike-in concentration from the SG-NEx AWS repository. To obtain sequin counts per million (CPM) levels, we followed the same method by Chen et al. [9]. The ground truth sequin CPM for a sequin transcript $x$ in a given sample $s$ was computed as follows:

13 $${\text{CPM}}_{x}=\frac{{a}_{x}}{{\sum }_{t\in T}{a}_{t}}*{c}_{s}*1000000$$

where $a$ is the set of raw abundances provided by SG-Nex, $t$ iterates through the entire set of transcripts to get the sum of all abundances, and ${c}_{s}$ is the spike-in concentration in sample $s$.

### Paired short- and long-read RNA-seq data

For humans, we employed paired short- and long-read RNA-seq data from the SG-NEx collection and long-read transcriptome profiling of human lung cancer cell lines data sets. Short- and long-read datasets are considered paired if they were obtained by sequencing the same biological sample. A subset of these samples included spike-in RNAs, and their reads were aligned to augmented versions of the GRCh38 genome that also includes the sequin-containing in silico chromosomes, provided by the original authors. All other samples (i.e., not spiked) were aligned to the regular GRCh38 p13 genome.

The goal with paired RNA-seq data sets is to compute the correlation between the short- and long-read-derived transcript abundance estimates. Long reads are first aligned to the GRCh38 genome using minimap2 and the resulting alignments are provided to StringTie for a transcriptome assembly. Short reads are then quantified on the long-read-derived StringTie transcripts using Salmon. Afterward, we ran quantification-only methods—NanoCount and TranSigner—on the StringTie assembly to obtain long-read-derived abundance estimates. We evaluated these tools’ estimates based on their nonlinear correlation with Salmon’s short-read-derived estimates (see Additional file: Note S3 for the commands used for short-read quantification). We repeated the same steps for two other organisms: *A. thaliana* and *M. musculus*. None of the samples from these two species contained sequins, so all reads were aligned to their respective reference genomes.

### Read assignments evaluation

For simulated and sequin data, we can define the following values based on the known origin transcript of each read:

- True positive (TP): a read is correctly assigned to its true origin.
- False positive (FP): a read is incorrectly assigned to a transcript that is not its true origin.
- False negative (FN): a read is not assigned to its true origin.

If a read is assigned to multiple transcripts without specifying the fraction allocated to each transcript, then the read is evenly distributed among those transcripts, with these fractions contributing to TP and FP values as appropriate. If the exact fraction of a read assigned to a transcript is provided, those fractions are used instead. These definitions of TP, FP, and FN are designed to evaluate how accurately assigned reads reflect their true origins. They are based on the assumption that when a tool assigns a read to multiple transcripts without specifying exact fractions, users will infer an even distribution across the transcripts—an assumption that may not always be accurate.

For each sample, the recall value of a method for the read-to-transcript assignment is calculated as the number of TPs divided by the total number of reads sequenced from that sample. The precision value is computed as the number of TPs divided by the sum of TPs and FPs. F1 score is defined as 2 * precision * recall/(precision + recall).

### Transcript abundance estimates evaluation

By default, TranSigner outputs read counts as its quantification estimates. The read count of a transcript $t$ (denoted as ${\text{rc}}_{t}$) is the sum of all positive read fractions assigned to transcript $t$, and the relative transcript abundance of $t$ (denoted as ${\rho }_{t}$) is equal to ${\text{rc}}_{t}$ normalized by the sum of all transcript read counts, ensuring that ${\sum }_{t\in T}{\rho }_{t}=1$. Note that in a long-read RNA-seq experiment, each read counts as a transcript, making the sum of the read counts equivalent to the total number of transcripts identified from the long-read data.

When needed, TranSigner’s read count estimates were converted to counts per million (CPM) estimates using the formula ${\text{CPM}}_{t}={\text{rc}}_{t}/l*{10}^{6}$ where $t$ is a transcript and $l$ is the total number of aligned reads. TranSigner also outputs read-to-transcript assignments where each read is assigned to one or more transcripts. More precisely, TranSigner outputs a list of transcripts to which a read $r$ is assigned along with the fraction of $r$ assigned to each transcript in that list, or the $\alpha$ estimates. These assignments can be used to compute coverage estimates for transcripts as ${\lambda }_{t}={}^{({\sum }_{r\in {R}_{t}}{\alpha }_{rt}*l(r))}\!\left/ \!{}_{l(t)}\right.$ where ${\alpha }_{rt}$ is the fraction of $r$ assigned to transcript $t$, ${R}_{t}$ is the set of reads whose fractions were assigned to $t$, and $l$ is a function that returns the length of a read or a transcript. Additionally, when evaluating transcript abundances for novel isoforms identified by different tools (e.g., Bambu, FLAIR), we used GffCompare to establish mappings between the novel isoforms and the ground truth reference transcripts. Only full intron chain matches (i.e., class_code “ = ”) were accepted as a valid mappings, while all other matches were discarded.

### Evaluation of tools capable of transcriptome assembly

We assessed the quality of assemblies generated by StringTie, Bambu, and FLAIR using the intron chain-level sensitivity and precision values computed by GffCompare [39].

We benchmarked each tool using random samples of the RefSeq annotation to observe how well the completeness of the guides impacts the accuracy of the assembled transcriptome and the simulated ONT data. Specifically, we randomly sampled a percentage of the origin transcriptome, referring to the set of transcripts from which a set of reads are simulated, to remove from RefSeq. The guides were sampled to contain 21 different percentages between 0 and 100% of the origin transcriptome with 5% increments. For each percentage, we independently sampled the guides three times, yielding 63 different guides per read set. StringTie, Bambu, and FLAIR were provided with the same guide annotations. Additionally, StringTie and Bambu were provided with the same minimap2 alignment results produced using the recommended options for processing ONT RNA-seq data (*-x splice -uf -k14* for direct RNA reads and *-x splice* for cDNA reads); FLAIR had its own align module (see Additional file 3: Note S5). Unlike StringTie and FLAIR which output an annotation containing only the identified expressed transcripts, Bambu outputs both expressed and unexpressed transcripts in the guide annotation. Therefore, for our evaluations, we removed any transcript that was assigned a zero read count from Bambu’s output.

## Supplementary Information

Additional file 1. Supplementary Tables S1– S16. Includes table descriptions.

Additional file 2. Supplementary Figures S1– S7.

Additional file 3. Supplementary Notes S1– S5.

## Data Availability

The A. thaliana and M. musculus datasets are available from the European Nucleotide Archive (ENA) under accession numbers PRJEB32782 [39] and PRJEB27590 [40]. Specific ENA sample accession IDs for each pair of short- and long-read data sets are made available in Additional file 1: Table S13. The SG-NEx samples containing spike-in RNAs are available from the project AWS open data registry (https://registry.opendata.aws/sgnex/). The LRGASP data sets are available from the ENCODE portal. Specific LRGASP ENCODE sample IDs can be found in Additional file 1: Table S12. The long-read benchmarking on the human lung cancer cell lines data sets are made available from Gene Expression Omnibus (GEO) under accession number GSE172421 [41]. The values used to generate plots in this manuscript are made available as Additional file 1: Tables S1 ~ S14. TranSigner is publicly available at https://github.com/haydenji0731/transigner and is also archived on Zenodo at 10.5281/zenodo.14676146. The software is licensed under the CC BY 4.0. All code used to generate all figures (either in the main manuscript or in the supplementary materials) and the scripts and data files (e.g., ground truths for simulated and sequin data) used for benchmarking are available in Zenodo at https://zenodo.org/records/14940529. The transcript abundances used for read simulation are also available at the same address. The simulated ONT datasets (both direct RNA and cDNA), along with the RefSeq transcriptome files (including both annotation and transcript sequence FASTA files), are available at: ftp://ftp.ccb.jhu.edu/pub/data/NA12878_simulated_ONT/.
