# Supplementary material for: Enhancing transcriptome expression quantification through accurate assignment of long RNA sequencing reads with TranSigner
Source: Genome Biol. 2025 Aug 28;26:257. doi: 10.1186/s13059-025-03723-2 (PMC12392579; doi:10.1186/s13059-025-03723-2)
Supplement: Supplementary file 2 — Additional file 2. Supplementary Figures S1– S7. [file 13059_2025_3723_MOESM2_ESM.docx]

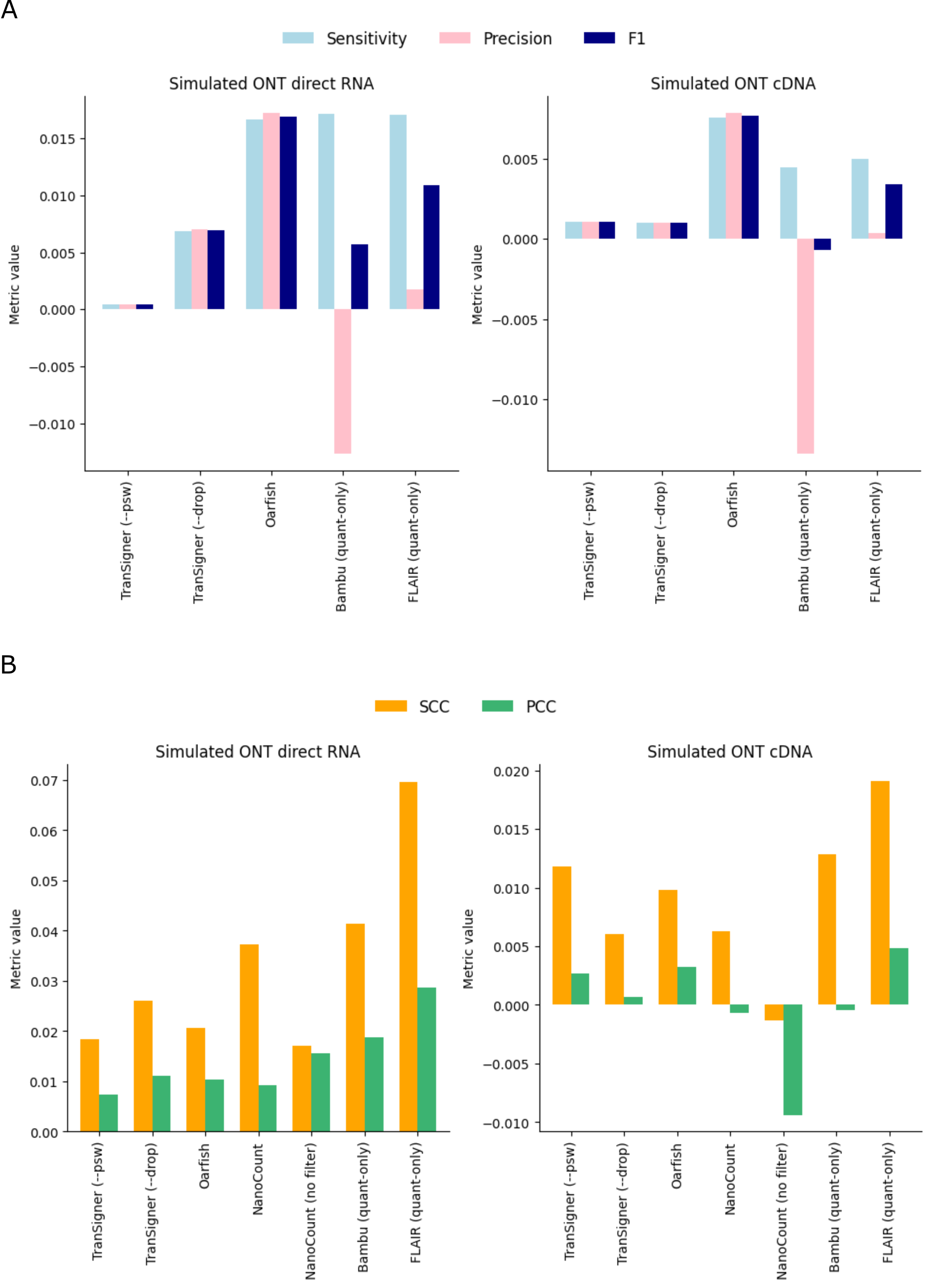


**Figure S1**. Changes in read assignment and quantification accuracies when the origin annotation provided in lieu of the full RefSeq annotation from which the reads were simulated. A: Average metric values evaluating read assignment accuracies including sensitivity, precision, and F1 scores, defined in the Methods section. B: Average metric values evaluating quantification accuracies through linear and nonlinear correlations (i.e,. SCC and PCC respectively) with the expected read counts.


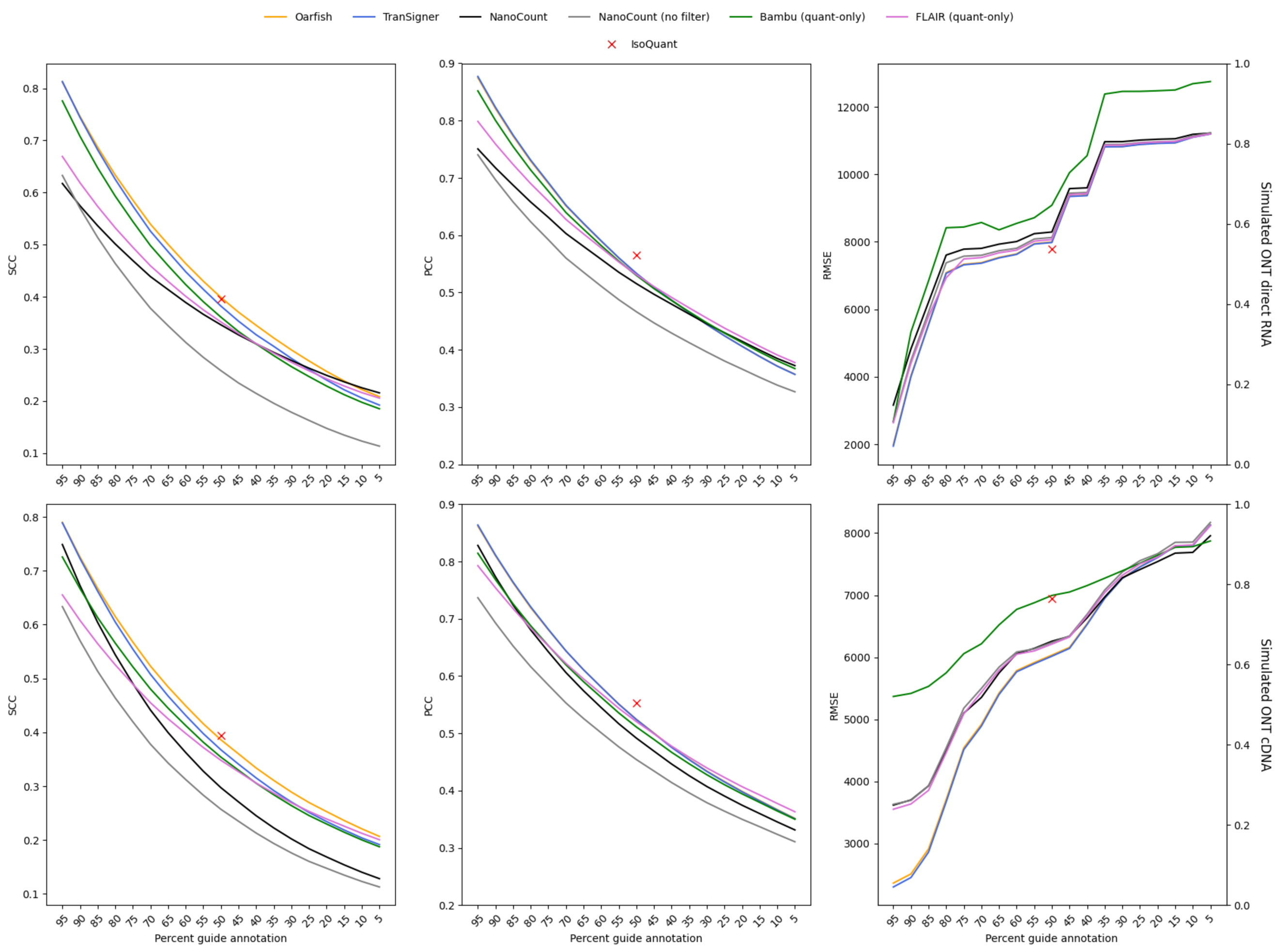


**Figure S2**. Average correlation coefficients and RMSE values computed by comparing true and estimated abundances computed at varying percent guide annotations computed when quantification-only methods are benchmarked on simulated ONT data (ONT direct RNA results on the top and ONT cDNA results at the bottom). Oarfish, TranSigner, NanoCount, NanoCount (no filter), Bambu (quant-only), and FLAIR (quant-only) were compared. IsoQuant was only run on 50% complete guides due to its excessive run times.


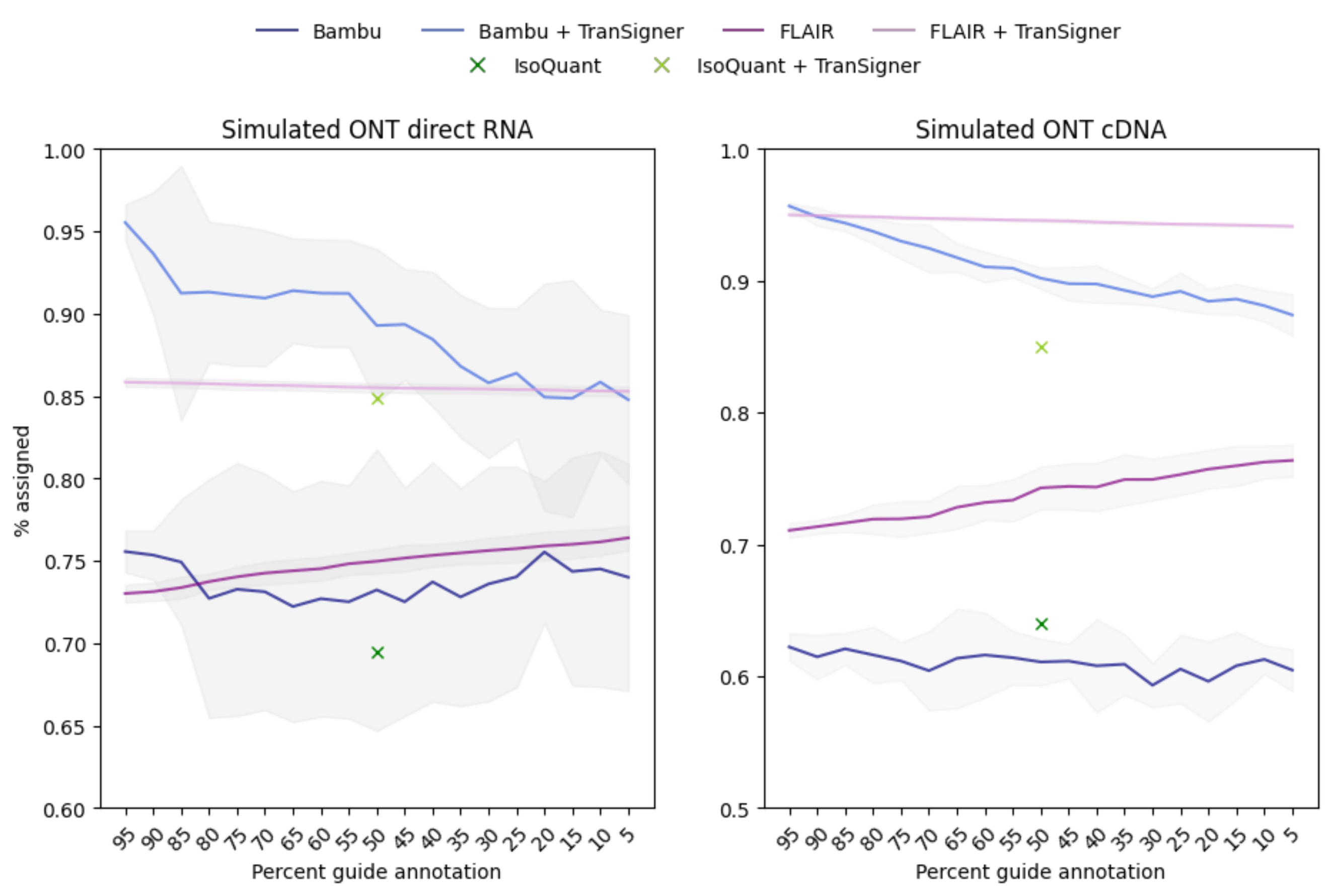


**Figure S3**. Percentage assigned observed for Bambu, Bambu + TranSigner, FLAIR, FLAIR + TranSigner. SD values are shown as shaded grey areas. IsoQuant and IsoQuant + TranSigner were only run on 50% complete guides due to the excessive IsoQuant run times.


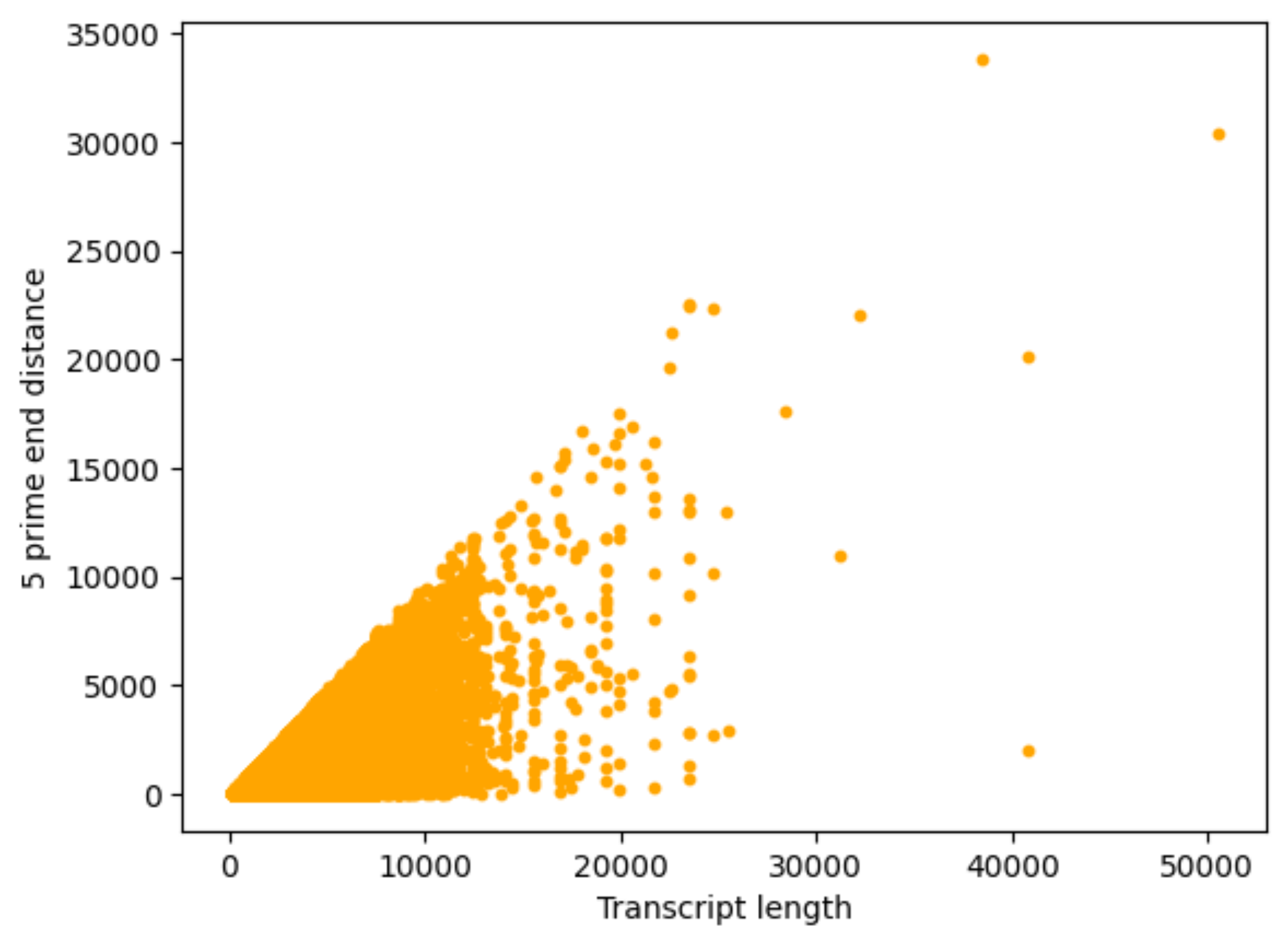


**Figure S4**. Scatter plot showing the correlation between transcript length and the 5’ transcript end distances for simulated ONT direct RNA reads aligned to the RefSeq reference transcripts. Only uniquely aligning reads were considered and end distances are indirectly measured as the alignment start positions computed by minimap2.

| **ALGORITHM 1: DROP** | | | | | |
| --- | --- | --- | --- | --- | --- |
|  | **Input**: a compatibility scores matrix $X$ and a matrix containing read fractions $\alpha$ | | | | |
|  | **Output**: updated compatibility scores matrix $X$ | | | | |
|  |  | | | | |
|  | for *r* = 1, … , *N* do (iterate through all reads in $\alpha$) | | | | |
|  |  |  | *M* ⃪ length($\alpha_{r}$) // $\vert\alpha_{r}\vert$ equal to $\vert T_{r}\vert$ in Methods  $k$ ⃪ 1 / *M* // $k$ equal to $\tau_{r}$ in Methods | | |
|  |  |  | for *t* = 1, … , *M* do (iterate through all transcripts aligned to a read) | | |
|  |  | |  | if $\alpha_{rt}$ < $k$ then | |
|  |  | |  |  | $X_{rt}$ ⃪ 0 |
|  |  | |  | else | |
|  |  | |  |  | do nothing |
|  |  | |  | end | |
|  |  | | end | | |
|  | end | | | | |
|  | | | | | |

**Figure S5.** Pseudocode for TranSigner’s drop algorithm. The input to this algorithm is two matrics, one storing the compatibility scores between reads and transcripts ($X$) and another containing the read fractions assigned to transcripts ($\alpha$). The output of this algorithm is the updated compatibility scores matrix $X$ that gets used to perform all subsequent E-steps, which isn’t shown above but described in Methods. A drop occurs when the fraction of a read $r$ assigned to a transcript $t$ is less than some threshold. It removes the compatibility relationship between a read $r$ and a transcript $t$ is removed by setting the $X_{rt}$ to 0 to ensure that $\alpha_{rt}$ remains 0 (i.e., no fraction of $r$ assigned to $t$) in subsequent E-step updates as it’s a product of $X_{rt}$. The drop threshold is dynamically computed for each read $r$ as 1 / $M$ where $M$ is the number of transcripts compatible with $r$.

| **ALGORITHM 2: PUSH** | | | |
| --- | --- | --- | --- |
|  | **Input**: a matrix storing the read fractions $\alpha$, and relative transcript abundances $\rho$ | | |
|  | **Output**: a list $\alpha^{'}$ containing hard assignments between reads and transcripts, new set of relative transcript abundances $\rho^{'}$ computed using those hard assignments | | |
|  |  | | |
|  | $\alpha^{'}$ ⃪ an empty list of size *N* (initialize $\alpha^{'}$) | | |
|  | for *i* = 1, … , *N* do (iterate through all reads) | | |
|  |  |  | *j* ⃪ discrete_sample($\alpha_{i})$ |
|  |  |  | $\alpha^{'}[i]$ ⃪ *j* |
|  | end | | |
|  | // perform another M-step to obtain the updated relative transcript abundances | | |
|  | $\rho^{'}$ ⃪ M_step($\alpha^{'}$) | | |
|  |  | | |

**Figure S6.** Pseudocode for TranSigner’s push algorithm that can be used to obtain 1-to-1 hard assignments between reads and transcripts. The input to this algorithm is a matrix containing read fractions assigned to transcripts ($\alpha$) and the relative transcirpt abundances ($\rho$). The output of this algorithm is a list containing hard read-to-transcript assignments ($\alpha^{'}$) and new relative abundances computed using these new assignments ($\rho^{'}$). The push algorithm assigns each read $r$ to the transcript with the most read fraction assigned, among all transcripts compatible with it. discrete_sample() function samples from a weighted discrete distribution defined by interpreting the read fractions $\alpha_{i}$ as weights for each discrete random variables.


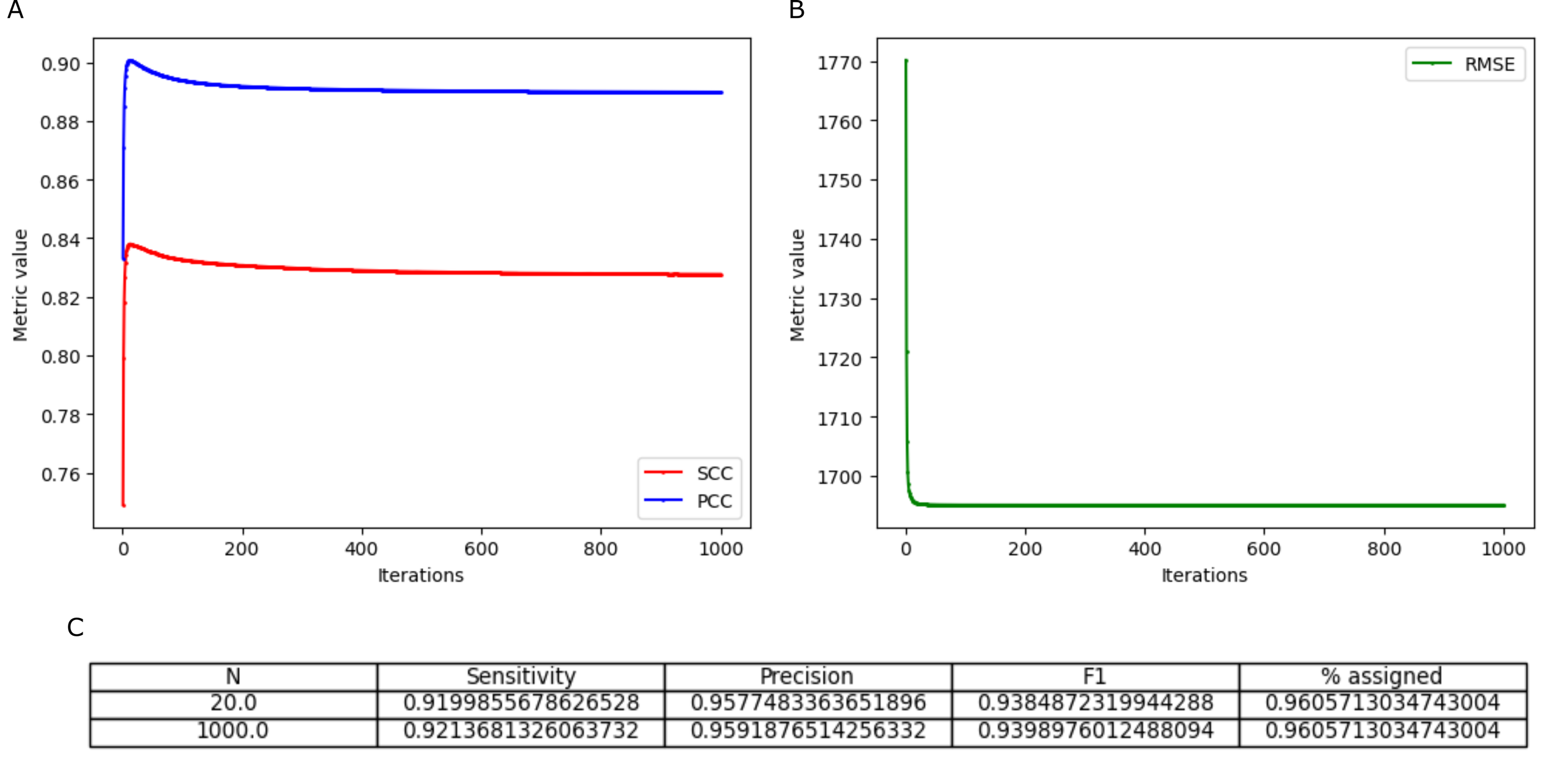


**Figure S7**. TranSigner’s abundance estimation and read assignment performances at varying EM iterations when benchmarked on a set of simulated ONT direct RNA reads and the full RefSeq annotation. A: scatter plot for the SCC and PCC values between the ground truth read counts and TranSigner’s abundance estimates across different EM iterations. B: scatter plot for the RMSE values between the ground truth read counts and TranSigner’s abundance estimates across different EM iterations. C: A table containing read assignment metric values–sensitivity, precision, F1, and % assigned–when TranSigner executed 20 and 1000 EM iterations. The N column contains the number of EM iterations executed. In order to force TranSigner to complete a specific number of iterations, we lowered the convergence threshold to an extremely small value and manipulated the maximum number of iterations parameter.
