# Supplementary material for: Enhancing transcriptome expression quantification through accurate assignment of long RNA sequencing reads with TranSigner
Source: Genome Biol. 2025 Aug 28;26:257. doi: 10.1186/s13059-025-03723-2 (PMC12392579; doi:10.1186/s13059-025-03723-2)
Supplement: Supplementary file 3 — Additional file 3. Supplementary Notes S1– S5. [file 13059_2025_3723_MOESM3_ESM.docx]

**Note S1. Short-read-based quantification commands.**

As described in Methods, we obtained short-read-based transcript abundance estimates on long read-derived StringTie transcriptomes using Salmon, given paired short and long-read sequencing data on the same biological sample. This analysis involved first running minimap2 to generate spliced genomic alignment of long reads. Different parameters were used for different read types (see Supplementary Text Table 2). Then, StringTie was used to obtain a long read-based transcriptome by running:

stringtie -L alignments.bam -o transcripts.gtf

Next, a Salmon index was created for this StringTie-assembled transcriptome by:

gffread -w transcriptome.fa -g genome.fa transcripts.gtf

cat transcriptome.fa genome.fa > gentrome.fa

salmon index -t gentrome.fa -d decoys.txt -i salmon_index

Note that the decoys.txt file was prepared as instructed in the Salmon documentation. Finally, a Salmon quantification was run as follows:

salmon quant -i salmon_index -l A -1 reads_1.fq -2 reads_2.fq -o salmon quant --validateMappings

**Note S2. Parameter tuning for 3’ and 5’ end distance thresholds**

TranSigner filters out the alignment between a read $r$ and a transcript $t$ if it starts and/or ends at a position far away from the 5’ and 3’ ends of $t$, indicating the aligned segment has a relatively small target cover. We defined 5’ end and 3’ end distances in the Methods section.

| $\boldsymbol{\beta}_{\boldsymbol{s}}$ **(-fp)** | $\boldsymbol{\beta}_{\boldsymbol{e}}$ **(-tp)** | **Spearman’s Correlation Coefficients (SCCs)** |
| --- | --- | --- |
| -550 | -300 | 0.6908945215530291 |
| -600 | -300 | 0.6913623708965158 |
| -550 | -400 | 0.6918556563135633 |
| -600 | -400 | 0.692299875863824 |
| -550 | -500 | 0.6923891562585597 |
| -600* | -500* | 0.6927918346209779 |
| **Supplementary Note Table 1. SCCs observed in the** $\boldsymbol{\beta}$ **parameter search on simulated ONT cDNA data.**  *TranSigner’s current recommended -fp and -tp parameters when processing ONT cDNA or PacBio samples. | | |

Based on the prior knowledge of frequent 5’ end truncations in ONT direct RNA reads, we set the 5’ end threshold, $\beta_{s}$, to a lenient value of -800, and no 3’ end filtering. Relatively little is known about the read distributions in ONT cDNA data, so we decided to tune the filter thresholds on a set of simulated ONT cDNA reads. We aimed to set the default values for $\beta_{s}$ and $\beta_{e}$ with ones that obtain the most accurate abundance estimates, as measured by their non-linear correlation with the known ground truth in the simulated ONT cDNA data. Briefly, we performed a grid search with $\beta_{s}$ values ranging from -550 to -600 with decrements by 50 and $\beta_{e}$ ranging from -300 to -500 with a decrement size of 100. We set $\beta_{s}$ to -600 and $\beta_{e}$ to -500 (see Supplementary Note Table 1).

Although varying the 5’ and 3’ end distance thresholds could slightly increase SCC values, we considered the improvement to be insignificant (observed maximum improvement ~ 0.0027). Therefore, we made these alignment end distance-based filters completely optional, as they do not substantially impact the tool’s performance. However, these optional filters allow users to eliminate highly unrealistic alignments based on their prior knowledge of where reads should align on transcripts.

**Note S3. ONT data simulation.**

We simulated ONT direct RNA and cDNA reads using NanoSim. We began the simulation by training a read profile using the NA12878 direct RNA and cDNA reads from Workman et al. using NanoSim’s read characterization module as follows:

read_analysis.py transcriptome -i reads.fq -rg genome.fa -rt reference.fa -annot reference.gtf -o out/na12878

We used the protein-coding and long non-coding transcripts in the RefSeq v110 annotation of GRCh38 to extract reference.fa and reference.gtf files. NanoSim also requires a quantification TSV file defining the abundances of transcripts to be expressed in the read set. We used Salmon, in its alignment-based mode, to obtain quantify transcript abundances in the Workman et al. NA12878 direct RNA and cDNA reads.

minimap2 -ax map-ont reference.fa reads.fq | samtools view -bS > alignments.bam

salmon quant -t reference.fa -l A -a alignments.bam -o salmon_quant --noErrorModel

We attempted to run Salmon in its --ont mode, but there were errors encountered so we used the --noErrorModel instead. We then extracted TPMs from the Salmon output to provide them for read simulation as follows:

simulator.py transcriptome -rt reference.fa -rg genome.fa -e salmon_tpms.tsv -c out/na12878 -o reads -n [14971421, 25418307] -r [dRNA, cDNA_1D2] --fastq

The number of reads specified (i.e., -n flag) corresponds to the number of reads in the Workman et al., NA12878 direct RNA and cDNA samples.

**Note S4. Long read data processing commands.**

We benchmarked Bambu (v3.2.5), FLAIR (v2.0.0), StringTie (v2.2.3), IsoQuant (v3.6.2), NanoCount (v1.1.0), Oarfish (v0.6.5), and TranSigner (v1.1.3) on simulated and experimental long read data. Note that StringTie and Bambu require an alignment file as an input, which we prepared using minimap2 (see Supplementary Text Table 2). With guide annotations, Bambu, StringTie, and IsoQuant were run as follows:

bambu(reads = alignments.bam, annotations = guide.gtf, genome = genome.fa, trackReads = TRUE)

stringtie -L alignments.bam -G guide.gtf -o transcripts.gtf

isoquant.py --reference genome.fa --genedb guide.gtf --bam alignments.bam --data_type nanopore -o out_dir

Without a guide annotation (i.e., in *de novo* mode), these three tools were run as follows:

bambu(reads = alignments.bam, annotations = NULL, genome = genome.fa, trackReads = TRUE, NDR = 1.0)

stringtie -L alignments.bam -o transcripts.gtf

isoquant.py --reference genome.fa --bam alignments.bam --data_type nanopore -o out_dir

Next, FLAIR was run as follows:

flair align -g genome.fa -r reads.fq --output flair.aligned

flair correct -q flair.aligned.bed -g genome.fa -f guide.gtf --output flair [--nvrna]

flair collapse -q flair_all_corrected.bed -g genome.fa -r reads.fq --output flair.collapse --gtf guide.gtf

flair quantify -r reads_manifest.tsv -i flair.collapse.isoforms.fa --output flair.quantify --generate_map

--nvrna flag was added when processing ONT direct RNA reads with FLAIR. Note that when processing experimental data, long RNA-seq reads were aligned to the genomes provided by those who generated the corresponding data sets (e.g., SG-NEx consortium, LRGASP).

Additionally, NanoCount was run on the minimap2-generated transcriptome alignments (see Supplementary Text Table 2) as follows:

nanocount [-n -d -1] -i alignments.bam -b sel_reads.bam --extra_tx_info -o tx_counts.tsv

Note that -n -d -1 flags were added when processing ONT cDNA reads with NanoCount, as specified in the tool documentation. Oarfish was run on the same set of alignments, but as name-sorted, using the following commands:

oarfish --alignments alignments.nsorted.bam --output prefix --filter-group no-filters --write-assignment-probs

Finally, TranSigner was run using the following set of commands executed consecutively:

transigner align -q reads.fq -t transcripts.fa -d out_dir

transigner pre -i alignments.bam -d out_dir

transigner em -s scores.csv -m tmap.csv -d out_dir -u unmapped.txt -dtype [ont_drna, ont_cdna, pacbio]

**Note S5. minimap2 alignment parameters**

The long-read analysis tools we benchmarked require that long reads are aligned to either the genome or the transcriptome of interest. StringTie, FLAIR, Bambu, and IsoQuant use genomic alignments, whereas NanoCount, Oarfish and TranSigner take in transcriptome alignments. FLAIR has its own align modules, so instead of running minimap2 ourselves, we ran their in-house module for benchmarking. For all others, we used the minimap2 parameters recommended by the original authors (see Supplementary Note Table 2).

| **ONT direct RNA** | |
| --- | --- |
| StringTie, Bambu, IsoQuant | minimap2 -ax splice -uf -k14 |
| NanoCount, Oarfish | minimap2 -ax map-ont -N 100 |
| TranSigner* | minimap2 -ax map-ont -N 181 |
| **ONT cDNA** | |
| StringTie, Bambu, IsoQuant | minimap2 -ax splice |
| **PacBio** | |
| StringTie | minimap2 -ax splice:hq -uf |
| Oarfish | minimap2 -ax map-pb -N 100 |
| **Supplementary Note Table 2. minimap2 parameters for genomic and transcriptomic alignments**  *TranSigner reuses the same minimap2 parameters for the transcriptomic alignment of all types of long reads. | |

As for transcriptomic alignments, NanoCount utilizes the minimap2 parameter preset for the alignment of genomic ONT reads but with an increased -N value. TranSigner uses an even higher -N parameter (i.e., 181) to retain all secondary alignments as described in Methods. 181 is the highest number of transcripts in a single gene locus according to the RefSeq release 110 annotation on the human GRCh38 genome. Users can adjust this parameter to an even higher number as needed, or provide a reference annotation to re-calculate the highest number of isoforms for a locus. Since RNA-seq reads are being aligned to fully processed transcripts (i.e., intron spliced out), the preset used for producing non-spliced, genomic alignments is employed. However, minimap2 parameters could be further optimized for transcriptomic alignment, which have different features than genomes.

StringTie, Oarfish, and TranSigner were further evaluated on PacBio data sets. We used the minimap2 presets recommended for processing PacBio IsoSeq and traditional cDNA data sets when benchmarking StringTie and Oarfish. As described before, TranSigner takes an approach that maximizes the number of secondary alignments, but we observed that minimap2’s PacBio preset yields far less alignments than its ONT preset. For that reason, we decided to employ the ONT preset for processing PacBio data sets as well. The quality of alignment is critical to the success during TranSigner’s EM stage, so further investigation on the optimal minimap2 parameters for transcriptomic alignments is called for.
